# Fuyuan Huoxue Decoction Promotes Lumbar Interbody Fusion in Rats: Role of Macrophage‐Driven Inflammation and the TLR9/NF‐κB Pathway

**DOI:** 10.1002/advs.78444

**Published:** 2026-10-29

**Authors:** Hongwei Gao, Han Wu, Shaorong Li, Weijian Wang, Yijian Guo, Shuai Cheng, Jiayi Sun, Jilong An, Wei Zhang

**Affiliations:** ^1^ Department of Spinal Surgery The Third Hospital of Hebei Medical University Shijiazhuang People's Republic of China; ^2^ Laboratory of Sensory Neurobiology School of Basic Medical Sciences Hebei University Baoding People's Republic of China; ^3^ School of Basic Medicine Hebei Medical University Shijiazhuang People's Republic of China; ^4^ Department of Spinal Surgery Affiliated Hospital of Hebei University Baoding People's Republic of China

**Keywords:** bone marrow mesenchymal stem cells, Fuyuan huoxue decoction, lumbar interbody fusion, macrophage‐to‐BMSC inflammatory signaling, osteogenic differentiation, osteoimmunology, TLR9/NF‐κB signaling

## Abstract

Delayed union and pseudarthrosis remain complications of lumbar interbody fusion. We investigated Fuyuan Huoxue decoction (FY) in a rat extreme lateral interbody fusion model. FY improved bone mineral density and trabecular architecture. Reduced inflammatory macrophage infiltration and nuclear factor kappa B (NF‐κB) activation at 4 weeks were accompanied by improved bony bridging at 8 weeks. Transcriptomic and network analyses nominated Toll‐like receptor 9 (TLR9)/NF‐κB signaling for functional investigation. FY‐medicated serum reduced macrophage inflammatory markers and extracellular double‐stranded DNA (dsDNA), promoted bone marrow mesenchymal stem cell (BMSC) osteogenesis, and attenuated impairment induced by inflammatory macrophage‐conditioned medium. DNase treatment reduced this medium's inhibitory effects, whereas a separate exogenous dsDNA challenge impaired osteogenesis and was attenuated by a TLR9 antagonist. TLR9 agonist and antagonist experiments in BMSCs and a 2‐week in vivo agonist intervention supported pathway involvement. Human nonunion single‐cell data showed increased phospholipid scramblase 1 and interleukin‐6 expression in myeloid cells. These findings support complementary pro‐osteogenic and anti‐inflammatory actions of FY and identify TLR9/NF‐κB as a candidate pathway contributing to fusion repair.

## Graphical abstract

Fuyuan Huoxue decoction improves lumbar interbody fusion in rats while reducing inflammatory macrophage activity and supporting bone‐forming stem cells. Experiments implicate extracellular DNA and TLR9/NF‐κB signaling in early responses. These findings point to coordinated control of inflammation and osteogenesis as a potential route to stronger spinal fusion.

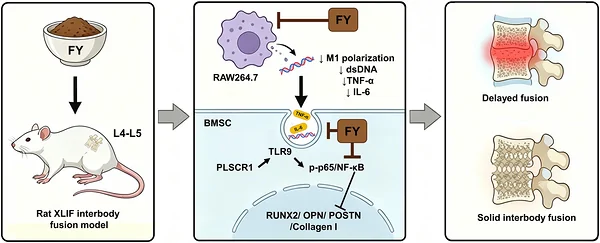

## 1 Introduction

The central goal of interbody fusion is to achieve a stable, continuous, and mechanically supportive bony bridge between adjacent vertebral bodies. Despite continual improvements in bone‐grafting materials, internal fixation and interbody cages, delayed union and pseudarthrosis remain major obstacles limiting postoperative outcomes, and may secondarily lead to disc‐space collapse, cage migration and persistent low back pain [1, 2]. A nationwide cohort study of 65,355 cases showed that the reoperation rate within 3 years after degenerative spinal fusion was approximately 4.5% (5.0%–5.3% for lumbar approaches), with pseudarthrosis being one of the principal indications for revision, and revision fusion yielding worse outcomes than the primary procedure [3, 4]. Patient‐ and surgery‐related factors such as smoking, diabetes, and the non‐use of osteoinductive agents further increase the risk of fusion failure [1, 5, 6], underscoring a particularly pressing need to improve fusion quality in high‐risk populations. In fact, interbody fusion does not depend solely on the osteoconduction provided by graft materials, but is a complex process jointly governed by local inflammation, angiogenesis, stem‐cell recruitment, and osteogenic mineralization; therefore, improving the local microenvironment of the fusion site may be a key link in enhancing fusion quality [7, 8].

At present, the choice of perioperative fusion‐promoting agents is limited [9, 10]. Teriparatide (PTH) is constrained by its narrow eligible population, the need for long‐term subcutaneous injection, and its high cost; although exogenous osteoinductive factors such as rhBMP‐2 can promote osteogenesis, they carry risks including ectopic ossification, severe local inflammation, and osteolysis [11, 12, 13]. Notably, whether based on parathyroid‐hormone signaling or bone morphogenetic protein, these agents are primarily used for their osteoanabolic effects; persistent inflammatory inhibition of osteogenesis remains an additional therapeutic consideration. Within the framework of osteoimmunology, bone formation and the immune system are extensively co‐regulated, and a sustained postoperative inflammatory microenvironment—driven largely by overactivated macrophages—acts as an active upstream suppressor rather than a passive bystander of osteogenesis [14]. Although recent immunomodulatory approaches have begun to couple macrophage reprogramming with bone regeneration, they have been explored using locally implanted scaffolds and hydrogels, leaving a gap for a systemically administered agent that simultaneously resolves inflammation and supports osteogenesis at the fusion site [15, 16, 17, 18, 19]. In this regard, multi‐component traditional Chinese medicine formulas, which act on multiple targets and levels rather than on a single osteogenic node, offer a rationale for investigating combined effects on inflammation and osteogenesis at the fusion site [20, 21, 22]. Therefore, exploring interventional strategies that combine osteogenic activity, modulation of the inflammatory microenvironment, and a favorable safety profile is of important clinical value.

Bone marrow mesenchymal stem cells (BMSCs) are the core source of new bone formation at the fusion site, capable of differentiating into osteoblasts and secreting osteogenic matrix such as type I collagen, osteopontin (OPN) and periostin (POSTN) [23]. However, postoperative trauma induces massive infiltration of inflammatory cells, and persistently overactivated macrophages secrete mediators such as TNF‐α, IL‐6, and NO that can directly inhibit BMSC osteogenic differentiation and matrix mineralization [24, 25]. At the level of innate immunity, Toll‐like receptors are regarded as a key link connecting tissue injury with impaired bone repair: TLR9 recognizes CpG DNA and DNA‐type damage‐associated molecular patterns (DAMPs) and thereby activates downstream NF‐κB, and phospholipid scramblase 1 (PLSCR1) has also been reported to participate in the regulation of TLR9 signaling [26, 27, 28, 29]. Given that postoperative tissue injury and macrophage activation can release abundant DNA‐type DAMPs, which may in turn induce inflammatory stress in BMSCs [30, 31, 32], it remains to be clarified whether an aberrant TLR9/NF‐κB‐mediated signaling exists between inflammatory macrophages and BMSCs, and whether it can serve as an interventional target for improving fusion quality [15, 16, 33, 34].

Fuyuan Huoxue decoction (FY), originating from the Yi Xue Fa Ming (Medical Inventions), is renowned for promoting blood circulation and removing blood stasis, reducing swelling and relieving pain; modern pharmacology has confirmed that it possesses anti‐inflammatory, analgesic, microcirculation‐improving, and tissue‐repair‐promoting activities [20, 35]. However, previous studies have mostly focused on postoperative pain relief and soft‐tissue repair, and whether FY can improve osteogenic dysfunction under an inflammatory microenvironment by regulating macrophage‐to‐BMSC inflammatory signaling, and thereby promote interbody fusion, remains unclear. In addition, most existing fusion‐promoting studies have used the rat posterolateral intertransverse model, in which osteogenesis occurs at the intertransverse periosteal–muscular interface and which lacks the limited blood supply, endplate interface, and axial loading unique to the intervertebral space, making it difficult to faithfully reflect clinical interbody fusion [8, 36]. For this reason, the present study employed a rat extreme lateral interbody fusion (XLIF) model, in which fusion was evaluated at the anatomically and mechanically authentic site following nucleus pulposus removal, cartilaginous endplate preparation, and autologous iliac‐bone interbody grafting.

Based on the above background, this study hypothesized that FY, by suppressing excessive macrophage inflammation and relieving TLR9/NF‐κB‐related inflammatory stress within BMSCs, restores their osteogenic differentiation and matrix deposition, thereby promoting lumbar interbody fusion. To test this hypothesis, we combined micro‐CT, histology, UHPLC‐MS/MS identification of serum‐absorbed constituents, network pharmacology and in vivo/in vitro transcriptomics, and used LPS‐induced macrophage‐conditioned medium to simulate the inflammatory microenvironment of the fusion site in vitro, so as to examine the role of the TLR9/NF‐κB pathway therein and to explore the involvement of PLSCR1 as a candidate upstream node, aiming to provide a basis for intervening in osteogenic dysfunction under an inflammatory microenvironment and for the perioperative application of Chinese medicine in interbody fusion.

## 2 Materials and Methods

### 2.1 Drugs, Reagents and Antibodies

Fuyuan Huoxue decoction (FY) formula granules were prepared by Shijiazhuang Yiling Pharmaceutical Co., Ltd. according to the prescription proportions recorded in the Yi Xue Fa Ming (Medical Inventions) using a standardized process; in this study, UHPLC‐MS/MS was used to characterize its chemical constituents and serum‐absorbed constituents (see Sections 3.2 and Figure 3 and Tables S3 and S4). Before experiments, the granules were dissolved in the appropriate solvent as required.

The traditional FY formula comprises eight herbs: Radix Bupleuri (Bupleurum chinense DC., Apiaceae, dried root, lot no. B2412006), Trichosanthis Radix (Trichosanthes kirilowii Maxim., Cucurbitaceae, dried tuberous root, B2412001), Angelica sinensis (Angelica sinensis (Oliv.) Diels, Apiaceae, dried root, B2410002), Carthamus (Carthamus tinctorius L., Asteraceae, dried corolla, B2506001), peach kernel (Prunus persica (L.) Batsch, Rosaceae, ripe seed, B2409001), licorice (Glycyrrhiza uralensis Fisch., Fabaceae, root and rhizome, B2412001), rhubarb (Rheum officinale Baill., Polygonaceae, dried root and rhizome, B2410001), and pangolin (Manis pentadactyla L., dried scales). Because the pangolin is a Class I nationally protected wild animal in China and its use is strictly regulated by law, the FY formula granules actually prepared and administered in this study did not contain pangolin and consisted only of the seven herbs Bupleurum, Trichosanthes, Angelica sinensis, Carthamus, peach kernel, licorice and rhubarb combined in a ratio of 5:3:3:2:3:2:10 (corresponding to prescription amounts of 15, 9, 9, 6, 9, 6 and 30 g, respectively); pangolin is listed only to present the traditional composition of the formula in full. All in vivo and in vitro data were based on this seven‐herb formula, and the study strictly complied with relevant regulations and the ethical requirements for wildlife protection and scientific research.

Main reagents were DNase I (RNase‐ and protease‐free; MCE, HY‐108882B), calf‐thymus DNA (T13592), lipopolysaccharide (LPS; Solarbio, L8880), ODN1826 (MCE, HY‐146245), ODN2088 (MCE, HY‐150738), teriparatide (Solarbio, T7080; CAS 52232‐67‐4), CCK‐8 (Solarbio, CA1210), ALP staining and activity kits (Beyotime, C3206/P0321S), Alizarin Red S staining solution (Beyotime, C0148S), an extracellular dsDNA quantification kit (Solarbio, P9740), and the Omni‐Easy Ready‐to‐Use BCA Protein Assay Kit (Cat. No. ZJ102; Epizyme, Shanghai, China). A protein‐free rapid blocking buffer was obtained from Servicebio (G2052‐500 mL). Reverse‐transcription/qPCR reagents, PVDF membranes, and other routine reagents were used according to the manufacturers’ instructions.

Primary antibodies were iNOS (HUABIO, ER1706‐89), TNF‐α (HUABIO, R1203‐1), collagen I (Proteintech, 14695‐1‐AP and 67288‐1‐Ig), RUNX2 (HUABIO, ET1612‐47), OPN (Proteintech, 30200‐1‐AP), POSTN (Proteintech, 66491‐1‐Ig), TLR9 (ABclonal, A14642SP), NF‐κB p65 (Affinity Biosciences, BF3002), phospho‐NF‐κB p65 (Ser536; Affinity Biosciences, BF3001), PLSCR1 (Proteintech, 11582‐1‐AP), IL‐6 (HUABIO, R1412‐2), F4/80 (HUABIO, HA721520) and GAPDH (Proteintech, 10494‐1‐AP). The POSTN antibody was Periostin Monoclonal Antibody (clone 1A11A3), and the RUNX2 antibody was Recombinant Rabbit Monoclonal Antibody [SD208‐0].

Secondary antibodies for Western blotting, immunofluorescence and immunohistochemistry were supplied by Abkine and Proteintech. Host species, applications, dilution ratios, and catalog numbers for all primary and secondary antibodies are listed in Table S1.

### 2.2 Experimental Animals and Ethics

A total of 78 male SD rats (2 months old, 250 ± 50 g, purchased from the Experimental Animal Center of Hebei Medical University) were used: 54 for the main dose‐response fusion study (4‐ and 8‐week time points) and the serum‐pharmacology procedures, and an additional 24 for the in vivo TLR9‐intervention experiment (four groups, n = 6 each; Section 2.15.2 and Figure 9). They were housed at the Animal Experiment Center of the Third Hospital of Hebei Medical University under a 12 h/12 h light–dark cycle with free access to food and water, and experiments began after 7 days of acclimatization. The animal experiments were approved by the Animal Ethics Committee of the Third Hospital of Hebei Medical University (Z2026‐019‐1) and were reported in accordance with the ARRIVE guidelines.

Forty‐eight rats were used in the main XLIF experiment and were randomly divided into four groups—XLIF, low‐dose FY (L‐FY), high‐dose FY (H‐FY), and the teriparatide positive control (PTH)—with 12 rats per group; in each group, 6 rats were euthanized at 4 and 8 weeks postoperatively for micro‐CT and histological analysis. An additional 6 rats were used for early mechanistic exploration (in vivo transcriptomics, identification of serum‐absorbed constituents and preparation of medicated serum); these also underwent L4–L5 XLIF surgery and were randomly divided into a blank vehicle group (W) and an FY‐treatment group (F), with 3 rats per group, receiving continuous intragastric administration for 14 days from postoperative day 1, after which blood and fusion‐site tissue were collected in the same batch following the final dose. Grouping was performed by a random‐number method, and imaging, histological, and statistical analyses were all conducted by blinded investigators.

### 2.3 Establishment of the Rat XLIF Model

The rat XLIF model established by our group was used [8]. Anesthesia was induced with 5% isoflurane and maintained with 2%, and anesthetic depth was monitored by tail‐, ear‐, and limb‐pinch responses. With the rat in the right lateral decubitus position, the surgical area was shaved, disinfected, and draped; the L4–L5 disc space was localized at the level of the iliac crest, and a curved incision was made in the left abdomen approximately 3–4 cm from the midline. The external oblique, internal oblique, and transversus abdominis muscles were sequentially separated to expose the quadratus lumborum and retroperitoneal fat, and after blunt dissection, the anterolateral margin of the L4–L5 disc was exposed.

The annulus fibrosus was incised, the nucleus pulposus was thoroughly removed, and the cartilaginous endplates were curetted until punctate bleeding. Cancellous bone fragments harvested from the ipsilateral ilium were trimmed into bone particles for interbody grafting, adequately filling the L4–L5 disc space without over‐distraction, followed by lateral fixation with a miniature titanium plate and screws fitted to the L4–L5 segment. After hemostasis, the wound was closed in layers and covered with a dressing; postoperatively, the rats received warming, analgesia, and penicillin‐class antibiotics to prevent infection, and neurological function, activity, feeding, and wound healing were observed daily.

### 2.4 Drug Intervention

FY dose selection was based on the clinical prescription and body‐surface‐area conversion using the Meeh‐Rubner approach. L‐FY and H‐FY were administered at 1.125 and 4.5 g/kg/day, respectively, from postoperative day 1 until the 4‐ or 8‐week endpoint. All FY doses in this study are expressed as total daily doses (g/kg/day), with each daily dose divided equally between two intragastric administrations given 8–12 h apart. The 2.25 g/kg/day regimen used for medicated‐serum preparation and early mechanistic analyses followed the same twice‐daily schedule (Sections 2.7 and 2.16.1). XLIF controls received an equal volume of vehicle. Teriparatide was administered subcutaneously at 40 µg/kg once daily until the corresponding endpoint.

### 2.5 Micro‐CT Scanning and Bone Microstructure Analysis

At 4 and 8 weeks postoperatively, 6 specimens per group underwent high‐resolution micro‐CT scanning (SkyScan 1176, Bruker, Germany; resolution 18 µm, 65 kV, 385 µA, exposure 340 ms/projection, 360° scan), with reconstruction using DataViewer and three‐dimensional visualization using CTvox. The L4–L5 interbody graft and fusion region were defined as the region of interest (ROI), and CTAn was used to quantify BMD, BV/TV, BS/BV, Tb. N, Tb. Th, and Tb.Sp.

### 2.6 Histology, Immunohistochemistry and Immunofluorescence Staining

Specimens were fixed in 4% paraformaldehyde for 48 h, decalcified in 10% EDTA, dehydrated, and embedded in paraffin, after which 5 µm serial sections were prepared. HE staining was used to observe new bone, fibrous tissue, and callus, and Masson staining to assess collagen deposition and bone‐matrix maturation. Immunohistochemistry/immunofluorescence was used to evaluate inflammation, NF‐κB activation, and osteogenic matrix deposition: F4/80+iNOS double labeling was used to observe the infiltration of F4/80^+^iNOS^+^ pro‐inflammatory macrophages, and *p*‐p65+Collagen‐I dual immunofluorescence to observe the association between NF‐κB activation and osteogenic matrix expression. After deparaffinization, rehydration, antigen retrieval, and blocking, sections were incubated with primary antibodies overnight at 4°C, followed by HRP‐ or fluorophore‐conjugated secondary antibodies; immunohistochemistry was developed with DAB, and immunofluorescence nuclei were counterstained with DAPI. Images were acquired under uniform parameters, and positive area or fluorescence intensity was quantified with ImageJ.

Quantification of histology and immunostaining: all immunohistochemical and immunofluorescence sections were stained in the same batch, and images were acquired by blinded personnel under identical exposure, gain, and other parameters; for each animal, 3 non‐overlapping random fields were taken, and their mean was used as the quantitative value for that animal, with the animal as the independent statistical unit. All quantification was performed in ImageJ with consistent thresholds across groups. For Masson staining, the manually delineated interbody fusion region on each section was used as the ROI, and the proportion of collagen‐positive (blue‐stained) area within the ROI was calculated; because in some sections newly formed bone trabeculae could also be stained blue by Masson, to avoid such false positives, quantification was restricted to this fusion ROI and excluded non‐target blue‐stained areas such as bone trabeculae. F4/80^+^iNOS^+^ pro‐inflammatory macrophages were counted as the number of double‐positive cells (cells/field) by dual‐channel colocalization; *p*‐p65 and Collagen‐I immunofluorescence were expressed as mean fluorescence intensity (IntDen/Area); for OPN and RUNX2 immunohistochemistry, the DAB channel was separated by color deconvolution, and the percentage of DAB‐positive area was calculated using a uniform threshold. The above indices were normalized to the mean of the XLIF group and expressed as fold change relative to XLIF; the 4‐week and 8‐week time points were analyzed independently, without pooled testing across time points.

### 2.7 Identification of the In Vitro Chemical Constituents and Serum‐Absorbed Constituents of FY

FY granules were accurately weighed and extracted with a water:acetonitrile:isopropanol (1:1:1, v/v/v) extraction solution; after vortexing, low‐temperature ultrasonication, centrifugation, and protein precipitation, the supernatant was collected and vacuum‐dried, then reconstituted in 50% acetonitrile and analyzed by UHPLC‐MS/MS to complete the identification of the in vitro constituents.

The identification of serum‐absorbed constituents and the preparation of medicated serum used the 6 auxiliary animals described in Section 2.2 (all of which underwent XLIF surgery). The F group received FY at 2.25 g/kg/day by intragastric administration for 14 days, with the total daily dose divided equally between two administrations; the W group received an equal volume of sterile ultrapure water. After the final dose, peripheral blood was collected at 0.5, 1, and 2 h, allowed to stand at room temperature for 15 min, and centrifuged at 4000 rpm for 20 min at 4°C to separate the serum, and equal volumes from the three time points were pooled. The pooled serum was divided into two portions: one, without inactivation, was used directly for UHPLC‐MS/MS analysis of serum‐absorbed constituents, and the other was used to prepare medicated serum (see Section 2.11). After blood collection, the animals were euthanized, and L4–L5 fusion‐site tissue was harvested for in vivo transcriptomic analysis (see Section 2.16.1).

### 2.8 UHPLC‐Q‐Exactive Orbitrap MS/MS Analysis

A Vanquish UHPLC coupled to a Q Exactive HFX high‐resolution mass spectrometer (Thermo Fisher Scientific, USA) was used, with a Waters HSS T3 column (100 mm × 2.1 mm, 1.8 µm). Mobile phase A was ultrapure water containing 0.1% formic acid, and B was acetonitrile containing 0.1% formic acid; the flow rate was 0.3 mL/min, column temperature 40°C, and injection volume 2 µL. Gradient: 0–1 min, A/B 100:0; 1–12 min, linear to 5:95; 12–15 min, 5:95; 15.1–17 min, returned to 100:0. Samples were placed in a 4°C autosampler and injected in random order, with QC samples evenly interspersed to monitor system stability.

The mass spectrometer used an electrospray ionization source (ESI) with simultaneous acquisition in positive and negative ion modes: sheath gas 40 arb, auxiliary gas 10 arb, ion‐spray voltage +3000 V/−2800 V, ion‐source temperature 350°C, and transfer‐tube temperature 320°C; the scan mode was Full‐ms‐ddMS2, with MS1 m/z 70–1050 Da at a resolution of 70 000 and MS2 at a resolution of 17 500.

Raw data were processed with Progenesis QI (Waters, Milford, USA) for baseline filtering, peak recognition, peak matching, retention‐time correction, and peak alignment to generate a data matrix. Compound identification was based on the in‐house traditional Chinese medicine MS2 database of Sanshu Biotech, integrating retention time, accurate molecular mass, MS2 fragment ions, and fragmentation rules, with a molecular‐mass error ≤10 ppm; the MS2 fragment score was used to evaluate matching reliability. Differential metabolites were screened using a t‐test combined with OPLS‐DA, with criteria of *p* < 0.05, VIP > 1, and fold change >1.5 or <0.67.

### 2.9 Network Pharmacology and Molecular Docking Analysis

Based on the serum‐absorbed constituents, potential targets were predicted using SwissTargetPrediction and PharmMapper [37], disease targets related to spinal fusion, nonunion, and macrophage polarization were obtained from GeneCards, OMIM, DisGeNET, and the Therapeutic Target Database, with gene names standardized via UniProt [38]. The intersection of drug and disease targets was imported into STRING to construct a PPI network (Homo sapiens, score > 0.7) [39], visualized in Cytoscape, and core targets were screened using the topological parameter degree, retaining nodes whose degree was greater than the median degree of the network [40], GO and KEGG enrichment analyses were performed on the common targets.

The three‐dimensional structures of candidate constituents were obtained from PubChem and converted to PDBQT using OpenBabel; the structures of TLR9 and NF‐κB p65 were taken from PDB 3WPG and 2RAM, respectively, and the receptors were used for docking after removal of water and co‐crystallized ligands, addition of hydrogens, and charge assignment. Molecular docking was performed with AutoDock Vina using identical parameters for all compounds [41], with chloroquine and helenalin serving as reference inhibitors for TLR9 and NF‐κB p65, respectively. The lowest‐binding‐energy conformation was used for interaction analysis, and protein–ligand interactions were displayed with Discovery Studio Visualizer.

### 2.10 Culture of rBMSCs and RAW264.7 Cells

Rat bone marrow mesenchymal stem cells (rBMSCs, ORCR131) were purchased from Aopeisai Biotechnology (Shanghai) Co., Ltd.; after recovery, they were seeded in complete medium, and the medium was changed every 2 days after adherence, with routine passaging. RAW264.7 cells were purchased from the Cell Bank of the Committee on Type Culture Collection of the Chinese Academy of Sciences (Shanghai, China). Both cell types were cultured in basal medium containing 10% fetal bovine serum and 1% penicillin‐streptomycin at 37°C and 5% CO_2_; rBMSCs were used at passages 3–5 in the logarithmic phase, and RAW264.7 cells in good condition and not over‐confluent were used for subsequent experiments.

### 2.11 Preparation of FY‐Medicated Serum and Blank Serum

Medicated serum was prepared by the serum pharmacology method, with serum source, blood collection, and separation steps identical to those in Section 2.7. FY‐medicated serum from different time points was pooled in equal volumes to eliminate concentration fluctuations, and blank serum was prepared in parallel by the same procedure. Serum used for cell experiments was heat‐inactivated in a 56°C water bath for 30 min, sterilized through a 0.22 µm filter, aliquoted, and stored at −80°C, avoiding repeated freeze‐thaw cycles. Before experiments, serum was thawed at 4°C and added to the culture system as required: the RAW264.7 inflammation model and the rBMSC osteogenic‐induction experiments used three final concentrations of 2.5%, 5%, and 10%, with the corresponding blank‐serum groups receiving the same volume fraction of blank serum; for the cell‐viability assay, an additional 15% group was included to evaluate the effect of high‐concentration medicated serum on viability.

### 2.12 RAW264.7 Inflammation Model, M1 Polarization Assay and Extracellular dsDNA Measurement

RAW264.7 cells were used to establish an LPS‐induced inflammation model: for qPCR, Western blot, and dsDNA assays, cells were seeded in 6‐well plates (3 × 10^5^ cells/well), and for immunofluorescence, in coverslip‐containing 24‐well plates (3 × 10^4^ cells/well), and treated after overnight adherence. The experiment comprised the CON, LPS, Blank, 2.5% FY, 5% FY, and 10% FY groups: the FY groups were pretreated with the corresponding concentration of medicated serum for 2 h, and the Blank group received an equal volume fraction of blank serum; except for CON, all groups were stimulated with LPS (100 ng/mL) for 12 h. After treatment, cells were collected for qPCR, Western blot, and immunofluorescence, and the supernatant was assayed for dsDNA. M1 polarization was assessed by qPCR for Nos2, Cd86, Il6, and Tnf, Western blot for iNOS and TNF‐α, and immunofluorescence for iNOS, TNF‐α, and IL‐6.

Extracellular dsDNA was measured with a PicoGreen kit (Solarbio, P9740) according to the manufacturer's instructions: culture supernatant cleared of debris by centrifugation was taken, and a 1× working solution was prepared on the same day by 1:200 dilution in TE buffer and stored protected from light; a standard curve was established by serial dilution of calf‐thymus DNA; samples or standards were mixed with an equal volume of working solution, incubated at room temperature in the dark for 5 min, and the fluorescence was measured (excitation 488 nm, emission 520 nm), with the dsDNA concentration (ng/mL) calculated from the standard curve.

### 2.13 Preparation of Inflammatory Macrophage‐Conditioned Medium and the rBMSC Osteogenic‐Injury Model

RAW264.7 cells were stimulated with LPS (100 ng/mL) for 12 h. For the LPS/FY‐CM group, cells were pretreated with 5% FY‐medicated serum for 2 h before LPS stimulation. Cells were then washed three times with PBS, fresh medium was added, and conditioned medium was collected after a further 24 h and centrifuged to remove cellular debris. Con‐CM was collected from unstimulated RAW264.7 cells using the same collection procedure. LPS‐CM and LPS/FY‐CM denote conditioned media from LPS‐stimulated macrophages without and with FY pretreatment, respectively. Each conditioned medium was mixed 1:1 (v/v) with osteogenic induction medium for the rBMSC experiments. Thus, FY treatment in the LPS/FY‐CM arm was applied to macrophages before medium collection; in the separate experiments described in Section 2.15.1, FY‐medicated serum was added directly to LPS‐CM‐exposed rBMSCs.

#### 2.13.1 DNase Treatment and Separate Exogenous dsDNA Challenge

DNase I (RNase‐ and protease‐free; MCE, HY‐108882B) was used to reduce extracellular DNA in the conditioned medium. rBMSC viability was evaluated by CCK‐8 on days 1 and 3 over the concentration ranges shown in Figure 7A, and residual extracellular dsDNA after DNase treatment was measured using the PicoGreen assay (Solarbio, P9740) described in Section 2.12. The conditioned‐medium experiment comprised Con‐CM, Con‐CM+DNase, LPS‐CM, LPS‐CM+DNase, and LPS/FY‐CM groups, each applied under osteogenic induction conditions. The Con‐CM+DNase group assessed the effects of DNase treatment in the absence of inflammatory conditioned medium. DNase I was used at 10 µg/mL in the conditioned medium and incubated at 37°C for 30 min. The treated medium was then mixed 1:1 (v/v) with osteogenic induction medium and applied directly to rBMSCs, without a separate DNase‐removal step or addition of a DNase inhibitor. Corresponding untreated conditioned media underwent the same incubation and dilution procedures. LPS/FY‐CM was generated using 5% FY‐medicated serum during macrophage pretreatment.

In a separate experiment, calf‐thymus dsDNA (T13592) was added to rBMSCs at a final concentration of 5 µg/mL under osteogenic induction. The groups were OI, OI+dsDNA, OI+dsDNA+ODN2088, and OI+ODN2088; ODN2088 was used at 5 µg/mL as described in Section 2.15.1. This experiment tested the response to exogenous dsDNA and was not a re‐addition of DNA to DNase‐treated conditioned medium. Readouts included PLSCR1, TLR9, p65/*p*‐p65 and osteogenic proteins by Western blotting, p65/Collagen‐I immunofluorescence, ALP activity at day 7 and ARS mineralization at day 14 (Figure 7). The qPCR panel in the conditioned‐medium experiment comprised Runx2, Postn, Plscr1, Nfkbia, Col1a1, and Tlr9.

### 2.14 RBMSC Osteogenic Induction, CCK‐8, ALP and ARS Assays

After seeding, rBMSCs were cultured in osteogenic induction medium, and LPS‐CM, blank serum, or FY‐medicated serum was added according to the grouping. The induction medium was based on complete medium supplemented with β‐glycerophosphate, ascorbic acid, and dexamethasone to final concentrations of 10 mm, 50 µg/mL, and 10 nm (1 mL, 50 µL, and 500 µL of stock solution per 50 mL), with medium changed every 2 days and the serum proportion kept consistent across groups. CCK‐8 was used to assess viability on days 1, 3, and 5 of treatment; ALP staining and activity assays were used to assess early osteogenic differentiation on days 7 and 14 of induction; ARS staining was used to assess mineralized nodules on day 14, semi‐quantified by dissolution in 10% cetylpyridinium chloride and measurement of absorbance at 562 nm. Western blot, qPCR, and immunofluorescence were used to detect the expression of Collagen‐I, RUNX2, and OPN.

### 2.15 TLR9 Functional Intervention Experiments

#### 2.15.1 In Vitro Intervention in rBMSCs

To verify the role of the TLR9/NF‐κB pathway in FY's improvement of rBMSC osteogenic dysfunction under an inflammatory microenvironment, functional interventions were performed with the TLR9 agonist ODN1826 and the antagonist ODN2088. rBMSCs were first treated with different concentrations of ODN1826/ODN2088, and viability was assessed by CCK‐8; based on viability and experimental stability, 5 µg/mL was selected as the working concentration. After rBMSCs were cultured to 70%–80% confluence, the medium was switched to osteogenic induction medium and cells were grouped as follows: OI, OI+LPS‐CM, OI+LPS‐CM+5% FY, OI+LPS‐CM+5% FY+ODN1826, and OI+LPS‐CM+ODN2088; ODN1826 was used to observe whether TLR9 activation could offset the protective effect of FY, and ODN2088 to observe whether TLR9 inhibition could mimic the osteogenesis‐promoting effect of FY.

During the intervention, ODN1826/ODN2088 was applied as a pretreatment 1 h before the addition of LPS‐CM and medicated serum, followed by co‐incubation. For short‐term signaling assays: p65 phosphorylation and nuclear translocation were detected at 1–2 h of treatment; TLR9, p65 and *p*‐p65 proteins and Tlr9 and Nfkbia transcripts at 24 h; and Collagen‐I, RUNX2 and POSTN proteins and Col1a1, Runx2 and Postn mRNA at 48 h. In the long‐term osteogenic‐induction experiment, induction medium containing LPS‐CM, medicated serum, blank serum, ODN1826 or ODN2088 was changed every 2 days with conditions kept consistent; ALP staining and activity were used to evaluate early differentiation, ARS to evaluate late mineralization, and p65/Collagen‐I dual immunofluorescence to observe the relationship between NF‐κB activation and osteogenic matrix expression.

#### 2.15.2 In Vivo TLR9 Intervention (FY × ODN1826)

To validate the TLR9/NF‐κB pathway in vivo, 24 male SD rats (same source, age, and housing as above; ethics approval Z2026‐019‐1) underwent XLIF surgery as described in Section 2.3 and were randomly assigned to four groups (n = 6 each): XLIF (vehicle), H‐FY, ODN1826, and H‐FY+ODN1826. H‐FY animals received intragastric FY at 4.5 g/kg/day, with the total daily dose divided equally between two administrations from postoperative day 1, identical to the main experiment; the TLR9 agonist ODN1826 (MCE, HY‐146245) was administered by intraperitoneal injection at 100 µg per rat per dose in sterile saline, once every 3 days for a total of five doses (postoperative days 1, 4, 7, 10 and 13), and vehicle groups received an equal volume of sterile saline by the same route and schedule. All animals were euthanized at 2 weeks postoperatively to evaluate early postoperative responses, and L4–L5 fusion‐site tissue was harvested. Early tissue repair and inflammatory and osteogenic responses were evaluated by HE and Masson staining, TLR9 immunohistochemistry, OPN and RUNX2 immunohistochemistry, and F4/80^+^iNOS^+^ and *p*‐p65^+^/Collagen‐I dual immunofluorescence (n = 6 per group). Data were analyzed by two‐way ANOVA (FY × ODN1826) with Tukey post hoc tests.

### 2.16 RNA‐seq and Bioinformatics Analysis

#### 2.16.1 In Vivo RNA‐seq of Fusion‐Site Tissue

The 6 early‐mechanistic‐exploration rats that had undergone XLIF surgery, described in Section 2.2, were selected for in vivo RNA‐seq. The W group received an equal volume of sterile ultrapure water, and the F group received FY at 2.25 g/kg/day by gavage for 14 consecutive days from postoperative day 1, with the total daily dose divided equally between two administrations. After the final dose and simultaneous blood collection, L4–L5 fusion‐site tissue (including the interbody graft region, adjacent endplates and surrounding newly formed repair tissue) was isolated, with 3 biological replicates per group, snap‐frozen in liquid nitrogen and stored at −80°C for RNA extraction. Total RNA was extracted with TRIzol, and its concentration and purity were assessed with NanoDrop; qualified samples underwent poly(A) mRNA enrichment, fragmentation, cDNA synthesis, end repair, adapter ligation, and PCR amplification to construct libraries; raw data were quality‐filtered to obtain clean reads that were aligned to the rat reference genome, with expression expressed as TPM. Differentially expressed genes were screened with DESeq2 (|log2FC| > 0.563, Q ≤ 0.05), and GO and KEGG enrichment was performed to analyze changes in pathways related to inflammatory response, macrophage polarization, ECM remodeling, and osteogenic repair [42].

#### 2.16.2 In Vitro rBMSC RNA‐seq

A total of 9 samples were assigned to three groups—CON, FY and LPS‐Sup—with 3 biological replicates each: CON was the routine osteogenic‐induction group, LPS‐Sup was the osteogenic induction combined with LPS‐conditioned medium group (i.e., the aforementioned washed LPS‐CM, retaining the LPS‐Sup label in the transcriptome), and FY was the osteogenic induction combined with 5% FY‐medicated serum group. Total RNA was extracted with TRIzol and assessed with NanoDrop. Qualified samples underwent Oligo(dT) magnetic‐bead enrichment of poly(A) mRNA and fragmentation, reverse transcription with random N6 primers to synthesize double‐stranded cDNA, followed by end repair, 3′ A‐tailing, adapter ligation, PCR amplification, and single‐stranded circularization to construct single‐stranded circular DNA libraries, which, after quality control, were sequenced by PE150 on the DNBSEQ platform (BGI, Shenzhen).

Raw data were quality‐controlled with SOAPnuke to remove adapter contamination and high‐N and low‐quality reads, yielding clean reads; these were first aligned with HISAT to the rat reference genome Rattus_norvegicus_10116.NCBI.GCF_015227675.2_mRatBN7.2.v2201, then aligned with Bowtie2 v2.4.5 to the reference gene set, and quantified (TPM) with RSEM v1.3.1 [43, 44]. Each group produced an average of 6.64 Gb of clean data, with an average alignment rate of 98.54%, and a total of 18,103 expressed genes were detected. Pearson correlation coefficients between samples were calculated from the expression matrix and combined with PCA to assess clustering and replicate consistency. Differentially expressed genes were analyzed with DESeq2 (|log2FC| > 0.583, Q ≤ 0.05), and GO and KEGG enrichment Q values were obtained by the R phyper hypergeometric test with FDR correction, with Q ≤ 0.05 considered significant enrichment.

#### 2.16.3 Secondary Analysis of Public Single‐Cell Transcriptome Data From Human Nonunion

To assess the clinical relevance of the TLR9/NF‐κB inflammatory module in humans, a secondary analysis was performed on a public single‐cell dataset of human nonunion (GEO accession GSE217792; Avin et al., 2023) [45], which contains bone‐marrow‐cavity tissue scRNA‐seq from 5 nonunion patients and 4 non‐fractured controls. The 10x matrices of each sample were read and merged with Scanpy; after quality control (retaining cells with 200–6000 genes and a mitochondrial proportion < 20%), library normalization and log transformation, highly variable genes were selected, followed by PCA, neighbor‐graph construction and Leiden clustering, with UMAP visualization, and monocyte/macrophage populations were identified by annotation with classical marker genes [46]. Within this population, using the per‐sample mean expression (pseudobulk) as the statistical unit, the Mann‐Whitney U test was used to compare the expression of PLSCR1, IL‐6, TLR9, NFKB1, and RELA between nonunion and controls, with *p* < 0.05 considered significant (results in Figure 10A–C).

### 2.17 Western Blotting

Cells or tissues were washed with pre‐cooled PBS and lysed in lysis buffer containing protease and phosphatase inhibitors, and protein concentration was measured by the BCA method. Equal amounts of protein were separated by SDS‐PAGE and transferred to PVDF membranes; after blocking, membranes were incubated with primary antibodies overnight at 4°C, and the next day with fluorophore‐conjugated secondary antibodies, and images were acquired with a near‐infrared fluorescence imaging system; band intensity was analyzed by ImageJ and normalized to GAPDH.

### 2.18 Quantitative Real‐Time PCR

Total RNA was extracted with TRIzol and its concentration and purity measured with a NanoDrop 2000; 1 µg was reverse‐transcribed to cDNA and amplified with a TB Green system (20 µL/reaction): pre‐denaturation at 95°C for 30 s, and 40 cycles (95°C for 5 s, 60°C for 30 s). Relative expression was calculated by the 2^−ΔΔCt method and normalized to GAPDH; the primer sequences are provided in Table S2.

### 2.19 Cellular Immunofluorescence Staining

Coverslips were washed with PBS, then fixed, permeabilized, and blocked, and incubated with primary antibodies overnight at 4°C: iNOS, TNF‐α, and IL‐6 for RAW264.7; RUNX2, Collagen‐I, and OPN for the rBMSC osteogenesis experiments; and p65 and Collagen‐I for the TLR9 intervention experiments. The next day, they were incubated with fluorescent secondary antibodies, counterstained with DAPI, and mounted with anti‐fade medium; images were acquired under identical parameters, and mean fluorescence intensity or integrated density/area was calculated with ImageJ.

### 2.20 Eight‐Week Tolerability Assessment

Tolerability was assessed in the animals assigned to the 8‐week endpoint of the main XLIF experiment (XLIF, L‐FY, H‐FY and PTH; n = 6 per group), without an additional animal cohort. Body weight was recorded from the start of the experiment through postoperative day 56. At the 8‐week endpoint, the heart, liver, spleen, lungs, and kidneys were collected and processed for hematoxylin and eosin (H&E) staining to examine overt histopathological abnormalities. These assessments were used to describe tolerability over the observed treatment period; constituent pharmacokinetics and toxicity beyond 8 weeks were not evaluated.

### 2.21 Statistical Analysis

Data were analyzed with GraphPad Prism 9.3.1 and expressed as mean ± SD. Normality was tested by the Shapiro‐Wilk test before parametric testing: data that were normally distributed with homogeneous variances were analyzed by one‐way or two‐way ANOVA with Tukey's post hoc test, and those that did not meet these criteria by the Mann‐Whitney U or Kruskal‐Wallis test. For Figure 7, comparisons against the LPS‐CM or OI+dsDNA reference group used one‐way ANOVA with Dunnett's post hoc test; the Con‐CM versus Con‐CM+DNase comparison used an unpaired t‐test, as specified in the figure legend. The Figure 9 intervention was analyzed using two‐way ANOVA with FY and ODN1826 as factors and Tukey's post hoc comparisons. For indicators with three ordered dose levels (XLIF → L‐FY → H‐FY), a linear‐trend test was additionally used to formally assess dose dependence. *p* < 0.05 was considered statistically significant. All cell experiments were independently repeated at least 3 times, and animal experiments used each animal as an independent statistical unit.

## 3 Results

### 3.1 FY Promotes Lumbar Interbody Fusion in Rats and Improves the Bone Microstructure and Local Inflammatory Microenvironment of the Fusion Site

#### 3.1.1 Micro‐CT Shows That FY Dose‐Dependently Improves the Bone Microstructure of the Interbody Fusion Region

At 4 and 8 weeks postoperatively, micro‐CT imaging of the L4–L5 segment showed more new bone and improved trabecular continuity in the FY and PTH groups than in XLIF controls, with more extensive bony bridging at 8 weeks (Figure 1A,B). The quantitative results supported improvements in bone mass and architecture, including increased BMD, BV/TV, and Tb. N and lower Tb. Sp where indicated by the time‐point‐specific comparisons (Figure 1C). The ordered XLIF→L‐FY→H‐FY comparisons showed significant trends for selected parameters, with the individual trend *p*‐values displayed in Figure 1C. BS/BV showed no significant trend, and changes in Tb. Th were limited. H‐FY generally showed larger improvements in bone‐mass measures than L‐FY, but this pattern did not extend uniformly to all parameters; in particular, Tb. N at 8 weeks was similar in the two FY groups. The trend tests support an overall dose‐related pattern but do not establish a significant H‐FY versus L‐FY difference for every outcome. H‐FY values approached those of PTH for several measures, without a formal equivalence claim. BMD and BV/TV increased further at 8 weeks compared with 4 weeks, as indicated by the within‐group time comparisons in Figure 1C.

**FIGURE 1:**
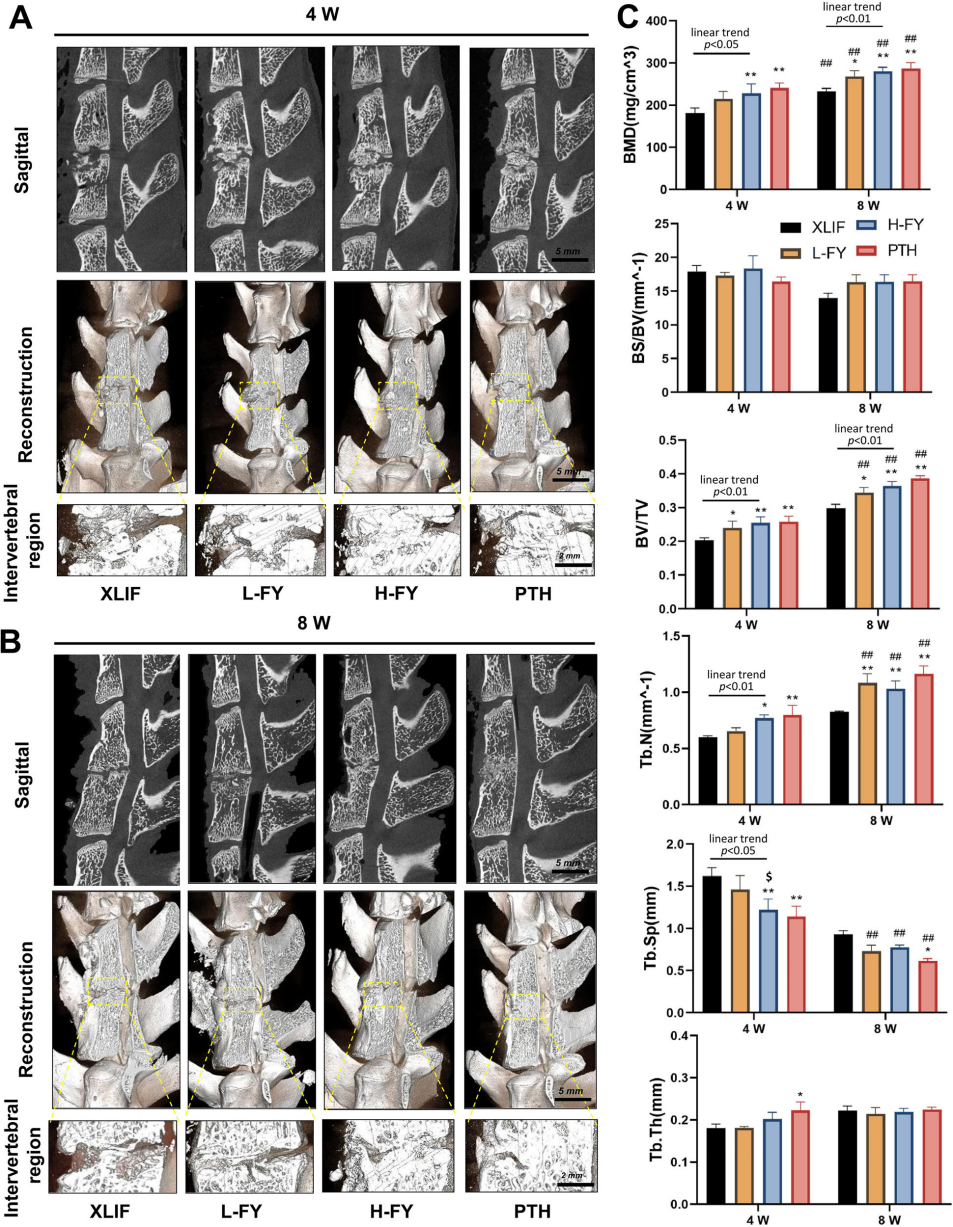
FY improves bone formation and microarchitecture in the rat XLIF fusion region. (A, B) Micro‐CT images at 4 and 8 weeks, respectively: sagittal views, three‐dimensional reconstructions and enlarged fusion regions. Scale bars: see the corresponding images in the main text. (C) Bone mineral density (BMD), bone surface/bone volume (BS/BV), bone volume/tissue volume (BV/TV), trabecular number (Tb.N), separation (Tb.Sp), and thickness (Tb.Th). Mean ± SD; n = 6 animals/group/time point. Ordered‐group trends (XLIF→L‐FY→H‐FY) are indicated. Two‐way ANOVA with Tukey comparisons: ^*^ *p* < 0.05, ^**^ *p* < 0.01 versus contemporaneous XLIF; #p < 0.05, ##p < 0.01 for 8 versus 4 weeks within each group; ns, not significant. FY, Fuyuan Huoxue decoction; XLIF, extreme lateral interbody fusion; L‐FY/H‐FY, FY at 1.125/4.5 g/kg/day, each daily dose divided equally into two administrations; PTH, teriparatide.

#### 3.1.2 The Histological Assessment Revealed That FY Can Promote New Bone Formation and Alleviate Inflammatory Responses

At 4 weeks postoperatively, HE staining showed that the fusion region in the XLIF group was dominated by fibrous‐like tissue and immature callus with sparse new trabeculae; after FY and PTH intervention, new bone increased, trabeculae were continuous, and local bony bridging was more evident (Figure 2A); Masson staining showed that after FY and PTH intervention the immature fibrous collagen filling the fusion gap was reduced, consistent with replacement of fibrous tissue by more mature bone (Figure 2C; the non‐trabecular collagen‐positive area quantified in Figure 2I was lower than in the XLIF group). Immunohistochemistry for osteogenic markers showed that OPN and RUNX2 positive expression was low in the XLIF group, and after FY and PTH intervention, the positive areas of both increased significantly, indicating that osteogenic matrix synthesis and osteogenic transcription‐factor expression were enhanced in parallel (Figure 2G, quantification in Figure 2M). Inflammation assays: F4/80^+^iNOS^+^ double labeling showed extensive infiltration of pro‐inflammatory macrophages in the XLIF group, whose signal was markedly attenuated after FY intervention; *p*‐p65^+^Collagen‐I dual immunofluorescence showed high NF‐κB activation and insufficient Collagen‐I in the XLIF group, and FY suppressed early NF‐κB activation and promoted osteogenic matrix deposition (Figure 2E; F4/80^+^iNOS^+^ counts in Figure 2J; *p*‐p65 and Collagen‐I in Figure 2L), with *p*‐p65 negatively correlated with Collagen‐I across animals (Pearson *r* = −0.68, Spearman *r* = −0.65, *p* < 0.0001; Figure 2K).

**FIGURE 2:**
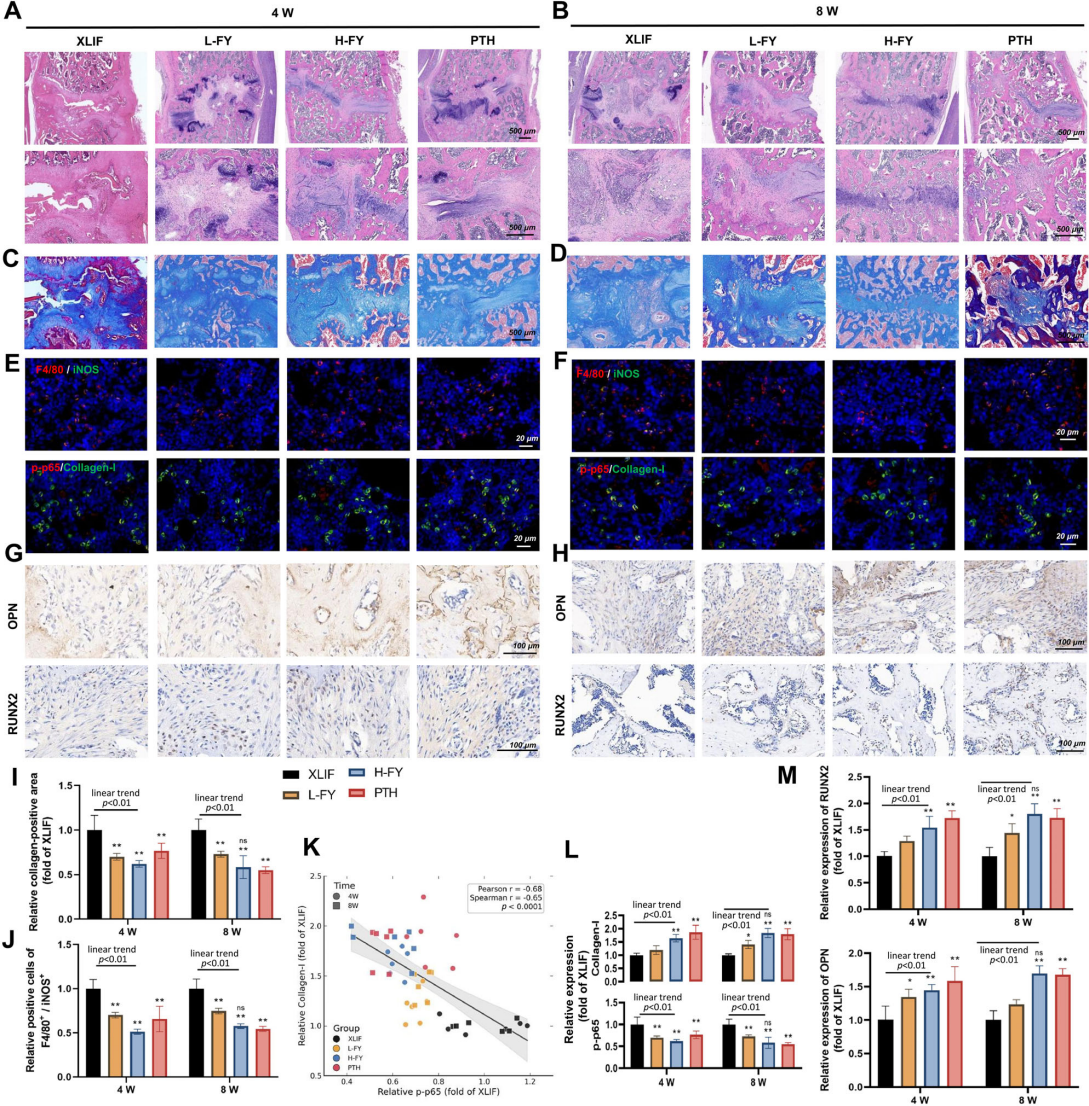
FY reduces inflammation and improves tissue repair at the fusion site. (A, B) H&E staining at 4 and 8 weeks, respectively, with enlarged boxed regions. (C, D) Masson staining. (E, F) F4/80/iNOS and p‐p65/Collagen‐I dual immunofluorescence. (G, H) OPN and RUNX2 immunohistochemistry. (I) Non‐trabecular Masson‐positive collagen area. (J) F4/80+iNOS+ cell counts. (K) Pearson and Spearman correlations between p‐p65 and Collagen‐I. (L) p‐p65 and Collagen‐I signals. (M) RUNX2‐ and OPN‐positive areas. Mean ± SD; n = 6 animals/group/time point. Histological readouts were normalized to contemporaneous XLIF controls, with time points analyzed separately. Ordered‐group trends are indicated; ^*^ *p* < 0.05, ^**^ *p* < 0.01 versus XLIF; ns, not significant. Scale bars: see the corresponding images in the main text.

At 8 weeks postoperatively, the above improvements were sustained and strengthened: HE and Masson staining showed more new bone and improved trabecular continuity in the FY and PTH groups, accompanied by a further reduction in the non‐trabecular fibrous collagen area as fibrous tissue was progressively replaced by bone (Figure 2B,D, quantification in Figure 2I); F4/80^+^iNOS^+^ macrophages maintained low infiltration and *p*‐p65 activation remained suppressed (Figure 2F; F4/80^+^iNOS^+^ counts in Figure 2J; *p*‐p65 and Collagen‐I in Figure 2L), while Collagen‐I was stably upregulated and OPN and RUNX2 remained higher than in the XLIF group (Figure 2H, quantification in Figure 2M). Notably, when FY reduced iNOS^+^ pro‐inflammatory macrophage infiltration and suppressed NF‐κB activation at 4 weeks, the bony bridge had not yet fully formed, and complete bony connection did not appear until 8 weeks, indicating that remodeling of the inflammatory microenvironment temporally preceded the completion of osteogenic fusion.

### 3.2 Serum Pharmacochemistry Identifies the Serum‐Absorbed Material Basis of FY

UHPLC‐MS/MS was used to analyze FY‐medicated serum in order to identify the serum‐absorbed active constituents. The total ion chromatograms in positive and negative ion modes showed good peak‐shape resolution and abundant signals, indicating a stable and reliable detection system (Figure 3A). Constituent classification showed that the serum‐absorbed constituents of FY were dominated by prenol lipids, flavonoids, organooxygen compounds, carboxylic acids and derivatives, and fatty acyls, along with isoflavonoids, coumarins, and steroids, indicating a chemically diverse serum‐associated constituent profile (Figure 3B; all identified constituents are listed in Table S3). PCA showed that the QC samples were highly clustered and the system was stable, and the FY and blank groups were completely separated (Figure 3C); the clustering heatmap of differential metabolites showed that numerous metabolites changed significantly after FY administration (Figure 3D).

**FIGURE 3:**
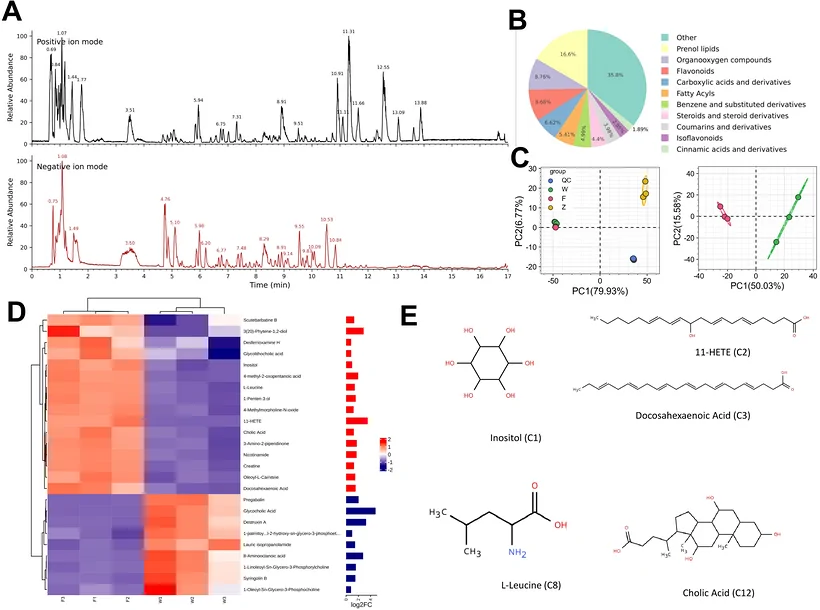
Chemical profiling of FY‐associated serum constituents. (A) Positive‐ and negative‐ion LC‐MS/MS total ion chromatograms. (B) Constituent classes. (C) Principal component analysis of serum profiles. (D) Differential‐constituent heatmap. (E) Representative structures: inositol (C1), 11‐HETE (C2), docosahexaenoic acid (C3), L‐leucine (C8), and cholic acid (C12). W, vehicle‐control serum; F, FY‐treated serum; Z, FY granules; QC, quality‐control samples. Constituent details are provided in Tables S3 and S4.

Further screening yielded 12 serum‐absorbed monomers, including inositol, 11‐HETE, docosahexaenoic acid, L‐leucine, creatine, nicotinamide and cholic acid (see Table S4); all 12 were used for network pharmacology and molecular docking analysis, and considering abundance, drug‐likeness and reported anti‐inflammatory/bone‐metabolism pharmacological background, 5 representative constituents (C1, C2, C3, C8, C12; correspondences in Table S4) were preferentially selected, with structures shown in Figure 3E. Among them, docosahexaenoic acid and cholic acid have a well‐defined anti‐inflammatory background and served as representative constituents for subsequent mechanistic analysis, while the contributions of individual constituents and possible interactions require further experimental evaluation.

### 3.3 Multi‐Omics Integration Suggests That FY's Action Converges on the Inflammatory (NF‐κB) and Matrix (ECM–Receptor) Modules

A cross‐integrated joint analysis was performed on three datasets: the in vivo transcriptome of the rat fusion region, the in vitro transcriptome of rBMSCs, and network pharmacology.

#### 3.3.1 In Vivo Transcriptome of the Rat Fusion Region: FY Remodels the Inflammation–Repair Balance

Compared with the blank group, a total of 861 differentially expressed genes were screened in the fusion region after FY intervention (459 upregulated, 402 downregulated; the volcano plot is shown in Figure 4A, and the DEG list is provided in Table S5). GO enrichment showed that the DEGs were mainly enriched in inflammatory response, cytokine‐mediated signaling, chemokine activity, ECM assembly, and tissue‐injury repair (Figure 4B); KEGG showed enrichment of ECM–receptor interaction, focal adhesion, TNF, NF‐κB, and IL‐17 pathways (Figure 4C and Table S6). The heatmap showed that FY downregulated pro‐inflammatory (M1‐like) marker genes such as S100a8, Lcn2, Retnlg and Il16 and upregulated anti‐inflammatory repair genes such as Arg1, Trem2, Gpnmb and Il10, remodeling the inflammation–repair balance of the fusion region in vivo (Figure 4D; the expression of osteogenesis/inflammation‐related genes was also assessed); GSEA further verified significant enrichment of matrix‐remodeling pathways such as ECM–receptor interaction and focal adhesion (Figure 4M).

**FIGURE 4:**
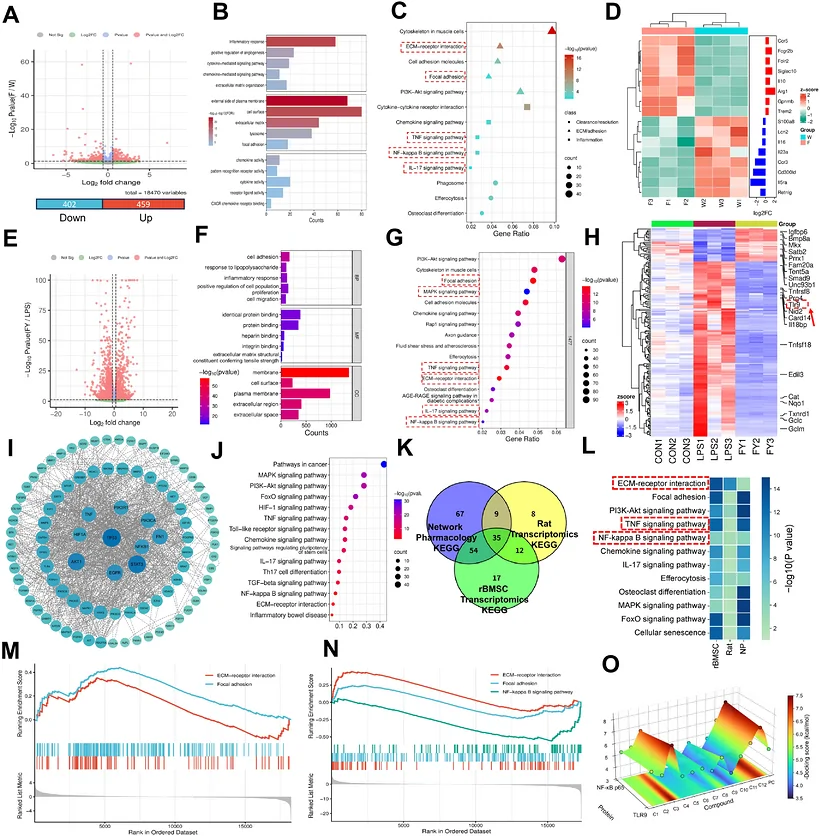
Integrated analyses nominate inflammatory and matrix‐related pathways. (A–D) Rat fusion‐site transcriptomics: differential‐expression volcano plot, GO enrichment, KEGG enrichment, and macrophage‐associated gene heatmap. (E–H) rBMSC transcriptomics: FY‐versus‐LPS‐Sup volcano plot, GO enrichment, KEGG enrichment, and selected gene‐expression patterns. The FY arm received osteogenic induction plus FY‐medicated serum without LPS‐CM. (I) Protein‐ protein interaction network. (J) Network‐pharmacology KEGG enrichment. (K) Shared pathways. (L) Twelve prioritized pathways. (M, N) In vivo and in vitro gene‐set enrichment analyses. (O) Predicted docking scores for 12 constituents against TLR9 and p65. n = 3 biological replicates/group. GO, Gene Ontology; KEGG, Kyoto Encyclopedia of Genes and Genomes.

#### 3.3.2 The In Vitro rBMSC Transcriptome Nominates TLR9 for Functional Investigation

PCA separated the CON, FY, and LPS‐conditioned‐medium groups, indicating distinct transcriptomic profiles under the three culture conditions. The DEG volcano plot is shown in Figure 4E; GO and KEGG enrichment overlapped highly with the in vivo transcriptome, being enriched in cell adhesion, inflammatory response, ECM composition, MAPK, TNF, NF‐κB, IL‐17, and ECM–receptor interaction pathways (Figure 4F,G and Table S8). The heatmap showed that FY upregulated osteogenic/matrix genes such as Igfbp6, Bmp8a, Satb2, Prrx1 and Smad9 while regulating oxidative‐stress genes such as Tlr9, Cat, Nqo1, Gclc and Gclm, indicating that FY concurrently modulates genes linked to inflammatory oxidative stress, ECM remodeling and osteogenic differentiation, and was therefore used to nominate candidate genes and pathways for functional validation (notably TLR9) rather than to quantify FY's effect on inflammation‐induced changes, which was instead addressed by the functional OI+LPS‐CM+FY experiments below (Figure 4H, list in Table S7); GSEA confirmed that ECM–receptor interaction, focal adhesion and the NF‐κB pathway participate in FY's regulation of rBMSCs (Figure 4N).

#### 3.3.3 Network‐Pharmacology Intersection Converges the Inflammatory Module Onto the TLR9/NF‐κB Axis

Based on the targets predicted for the 12 serum‐absorbed constituents of FY, intersection with disease targets related to spinal fusion, nonunion, and macrophage polarization yielded 105 core common targets (the constituent targets are listed in Table S9). Intersection of the KEGG pathways of the three datasets yielded 35 common pathways, with ECM–receptor interaction, focal adhesion, PI3K‐Akt, TNF, NF‐κB and IL‐17 stably enriched (Figure 4K and Table S10); the core‐target PPI network showed that NFKB1, TLR4, TNF, STAT3 and TP53 occupied core regulatory nodes (Figure 4I), with their KEGG pathway enrichment shown in Figure 4J and the top 12 pathways by correlation confidence in Figure 4L.

The integrated analyses highlighted NF‐κB‐associated inflammatory pathways and ECM receptor interaction/focal‐adhesion pathways related to matrix regulation. The in vitro transcriptome nominated Tlr9 as a candidate for functional investigation because its expression differed among the CON, LPS‐Sup, and FY conditions (Figure 4H). The FY RNA‐seq group received osteogenic induction plus FY‐medicated serum without LPS‐CM. Consequently, the FY versus LPS‐Sup comparison contrasts different treatment backgrounds and cannot establish that FY reverses or attenuates inflammation‐induced transcriptional changes. These data were used to identify candidate genes and pathways, whereas protection under inflammatory conditions was evaluated in the separate OI+LPS‐CM+FY functional experiments (Figure 8). PLSCR1 was investigated as a candidate upstream regulator based on its reported association with TLR9 signaling and its expression pattern; its functional role was not established by the transcriptomic analysis.

### 3.4 Molecular Docking Suggests That FY Serum‐Absorbed Constituents Have the Potential to Bind TLR9 and NF‐κB p65

AutoDock Vina predicted favorable docking scores for several candidate constituents against TLR9 and NF‐κB p65 (Figure 4O and Table S11). For C1, C2, C3, C8 and C12, the TLR9 scores were ‐5.8, ‐7.5, ‐7.5, ‐5.5 and ‐7.2 kcal/mol, respectively; the corresponding p65 scores were ‐5.3, ‐5.5, ‐6.7, ‐4.7 and ‐7.5 kcal/mol. Predicted poses and interaction maps identified possible hydrogen‐bonding, aromatic, and hydrophobic contacts within the modeled pockets. These computational results suggest potential interactions and support candidate prioritization, but do not measure binding affinity, demonstrate stable binding or establish direct target engagement.

### 3.5 FY‐Medicated Serum Inhibits LPS‐Induced Macrophage M1 Polarization and Reduces Extracellular dsDNA Accumulation

An M1‐like pro‐inflammatory model (characterized by upregulation of Nos2, Cd86, Il6 and Tnf) was constructed by stimulating RAW264.7 cells with 100 ng/mL LPS for 12 h, with pretreatment for 2 h with 2.5%, 5% and 10% FY‐medicated serum, respectively (Figure 5A). qPCR showed that Nos2, Cd86, Il6, and Tnf mRNA were significantly elevated in the LPS group, and FY‐medicated serum dose‐dependently downregulated these genes, whereas the blank‐serum group did not differ from the LPS group (Figure 5B). Western blot showed that LPS significantly upregulated iNOS and TNF‐α, which were progressively reduced with increasing FY concentration (Figure 5C,D); immunofluorescence was consistent, with enhanced iNOS, TNF‐α, and IL‐6 fluorescence in the LPS group that was dose‐dependently attenuated by FY (Figure 5F,G). For extracellular dsDNA, LPS significantly increased supernatant dsDNA, which FY dose‐dependently reduced, in a trend completely parallel to the downregulation of pro‐inflammatory factors (Figure 5E).

**FIGURE 5:**
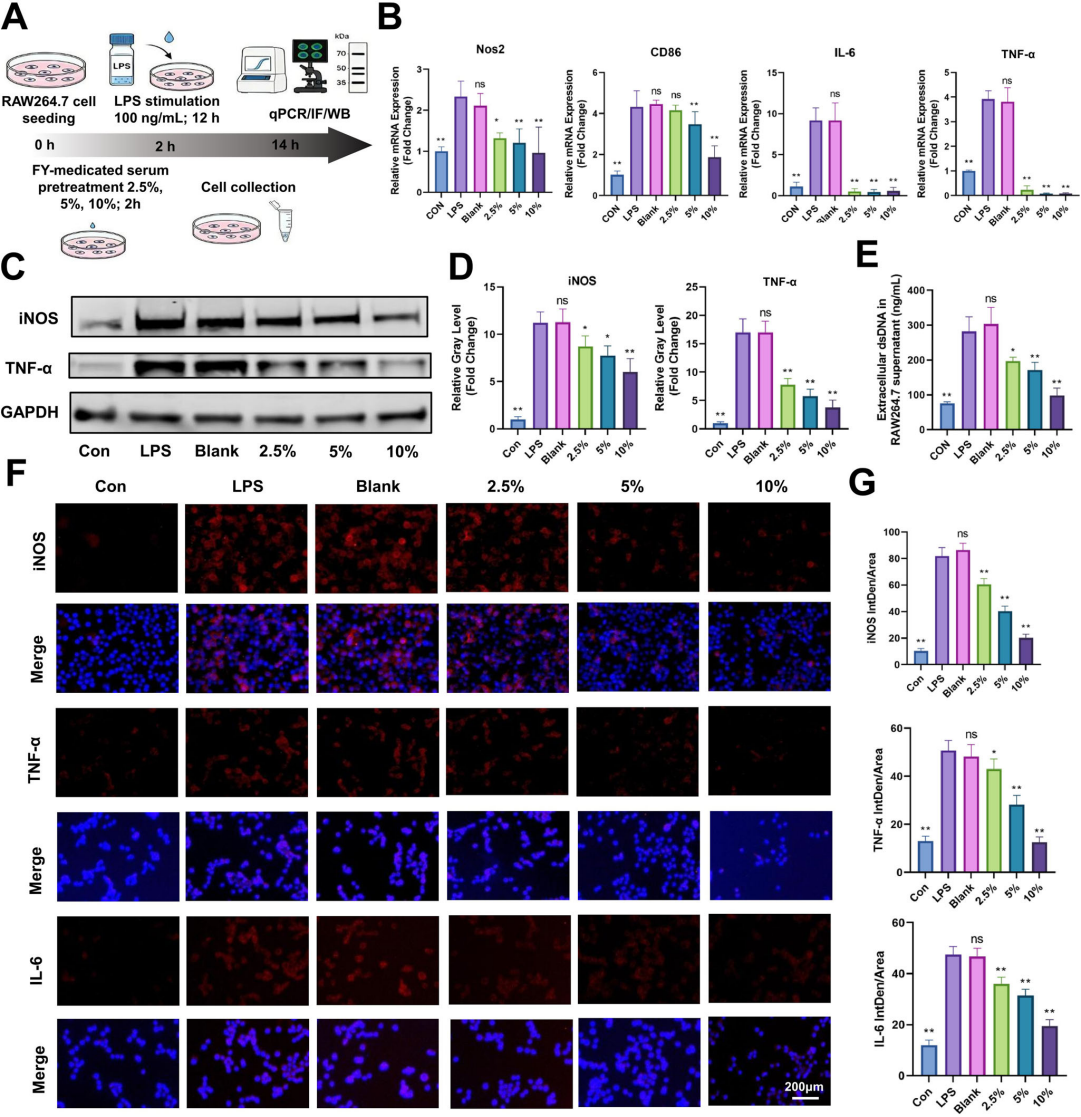
FY‐medicated serum reduces macrophage inflammatory markers and extracellular dsDNA. (A) FY‐serum pretreatment for 2 h followed by LPS stimulation for 12 h. (B) Nos2, Cd86, Il6, and Tnf transcripts. (C, D) iNOS and TNF‐α immunoblots and quantification. (E) Extracellular double‐stranded DNA (dsDNA). (F, G) iNOS, TNF‐α and IL‐6 immunofluorescence and quantification. Mean ± SD; n = 3 independent experiments. ^*^ *p* < 0.05, ^**^ *p* < 0.01, ^***^ *p* < 0.001 versus LPS; ns, not significant. Scale bars: see the corresponding images in the main text. Con, untreated control; LPS, lipopolysaccharide; Blank, blank‐serum control; percentages denote FY‐medicated‐serum volume fractions.

### 3.6 FY‐Medicated Serum Directly Promotes BMSC Osteogenic Differentiation and Mineralization

rBMSCs were treated with different concentrations of FY‐medicated serum, and viability, osteogenic markers, ALP activity, and mineralization capacity were assessed under an osteogenic‐induction system (the medicated‐serum preparation workflow is shown in Figure 6A). CCK‐8 showed no cytotoxicity and a mild pro‐proliferative effect at concentrations of 2.5%–15% on days 1, 3 and 5 of treatment (Figure 6B). ALP staining showed that on days 7 and 14 of induction, ALP activity in the FY groups increased progressively with concentration (Figure 6C, quantification in Figure 6E); on day 14 of induction, ARS showed that the number and staining intensity of mineralized nodules in the FY groups were significantly higher than in the blank and blank‐serum groups (Figure 6D, quantification in Figure 6E). qPCR showed that FY dose‐dependently upregulated Opn, Runx2, Ocn and Col1a1 (Figure 6F); Western blot showed that FY dose‐dependently upregulated Collagen‐I, RUNX2 and OPN, with no difference between the blank‐serum and blank groups (Figure 6G,H); immunofluorescence showed significantly enhanced RUNX2, Collagen‐I and OPN signals, consistent with the Western blot and qPCR results (Figure 6I,K, quantification in Figure 6L).

**FIGURE 6:**
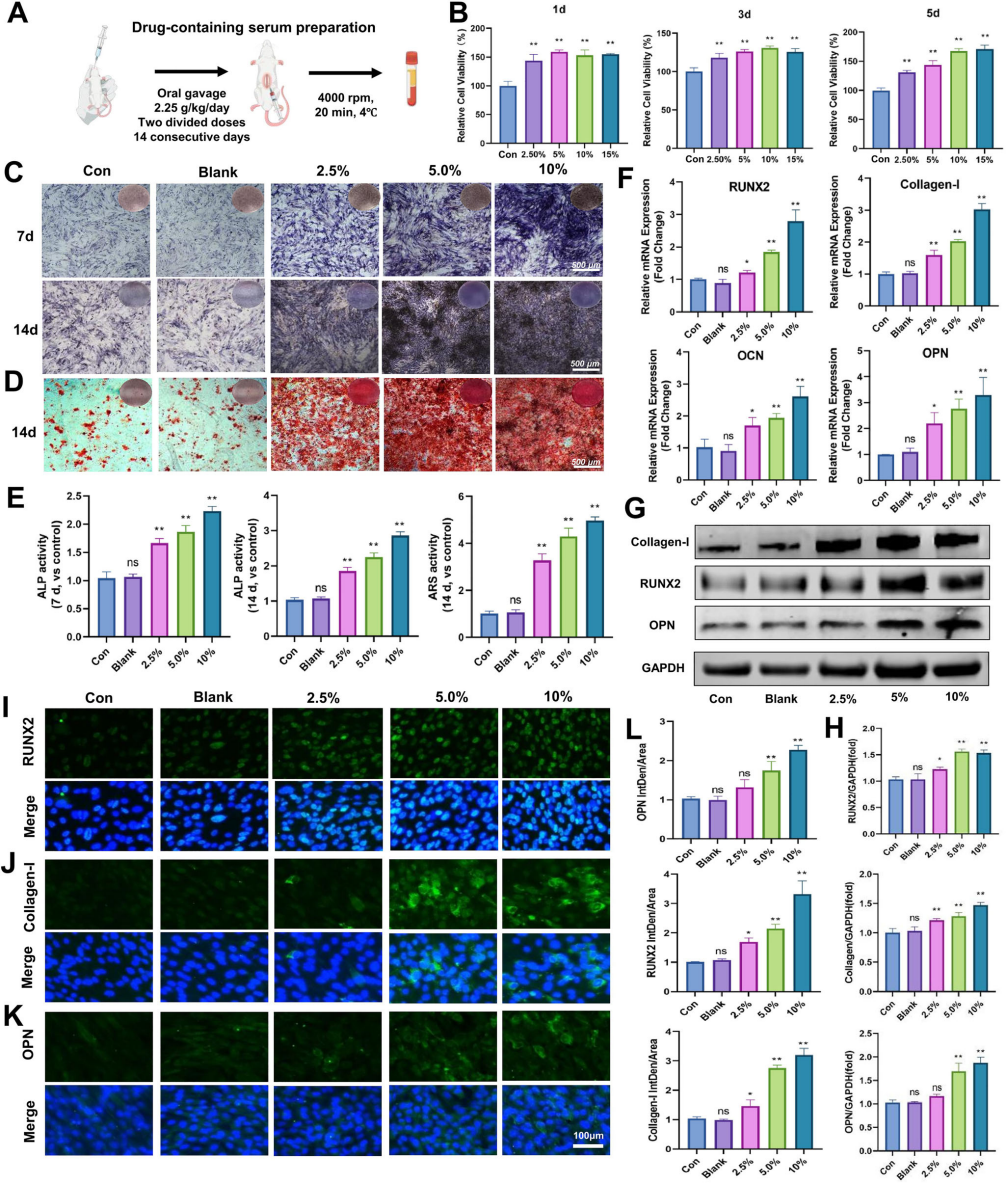
FY‐medicated serum promotes rBMSC osteogenesis under non‐inflammatory conditions. (A) Serum preparation: FY at 2.25 g/kg/day for 14 days, with each daily dose divided equally into two administrations. (B) CCK‐8 assays on days 1, 3, and 5. (C) Alkaline phosphatase (ALP) staining on days 7 and 14. (D) Alizarin Red S (ARS) staining on day 14. (E) ALP and ARS quantification. (F) Opn, Runx2, Ocn, and Col1a1 transcripts. (G, H) Collagen‐I, OPN and RUNX2 immunoblots and quantification. (I–K) RUNX2, Collagen‐I and OPN immunofluorescence, respectively. (L) Fluorescence quantification. Mean ± SD; n = 3 independent experiments. ^*^ *p* < 0.05, ^**^ *p* < 0.01 for the indicated comparisons; ns, not significant. Scale bars: see the corresponding images in the main text. Con, osteogenic‐induction control; Blank, blank‐serum control; percentages denote FY‐medicated‐serum volume fractions.

### 3.7 DNase Treatment and Exogenous dsDNA Challenge Support a Contribution of Extracellular DNA to Inflammatory Osteogenic Impairment

To investigate whether extracellular DNA contributes to the inhibitory effects of inflammatory macrophage‐conditioned medium, we combined DNase treatment with a separate exogenous dsDNA challenge. CCK‐8 assays evaluated the concentration‐dependent effects of DNase and dsDNA on rBMSC viability (Figure 7A); the working dsDNA concentration was 5 µg/mL. DNase treatment reduced extracellular dsDNA in the conditioned medium (Figure 7B,C). The Con‐CM, Con‐CM+DNase, LPS‐CM, LPS‐CM+DNase, and LPS/FY‐CM groups were then compared under osteogenic induction. Relative to LPS‐CM alone, DNase‐treated LPS‐CM showed attenuated TLR9/NF‐κB‐associated responses and improved expression of osteogenic markers, ALP activity, and ARS mineralization. LPS/FY‐CM, collected after FY pretreatment of macrophages, also reduced these inhibitory effects (Figure 7D–H). In the separate OI, OI+dsDNA, OI+dsDNA+ODN2088 and OI+ODN2088 experiments, exogenous dsDNA increased TLR9/NF‐κB‐associated responses and impaired osteogenesis, and ODN2088 attenuated these changes (Figure 7I–M). Together, these results support a contribution of extracellular DNA to the activity of LPS‐CM and pharmacological involvement of TLR9 in the response to exogenous dsDNA. Because DNA was not re‐added to DNase‐treated LPS‐CM, the experiments do not establish that macrophage‐derived dsDNA is the specific or sole mediator of the conditioned‐medium effect.

**FIGURE 7:**
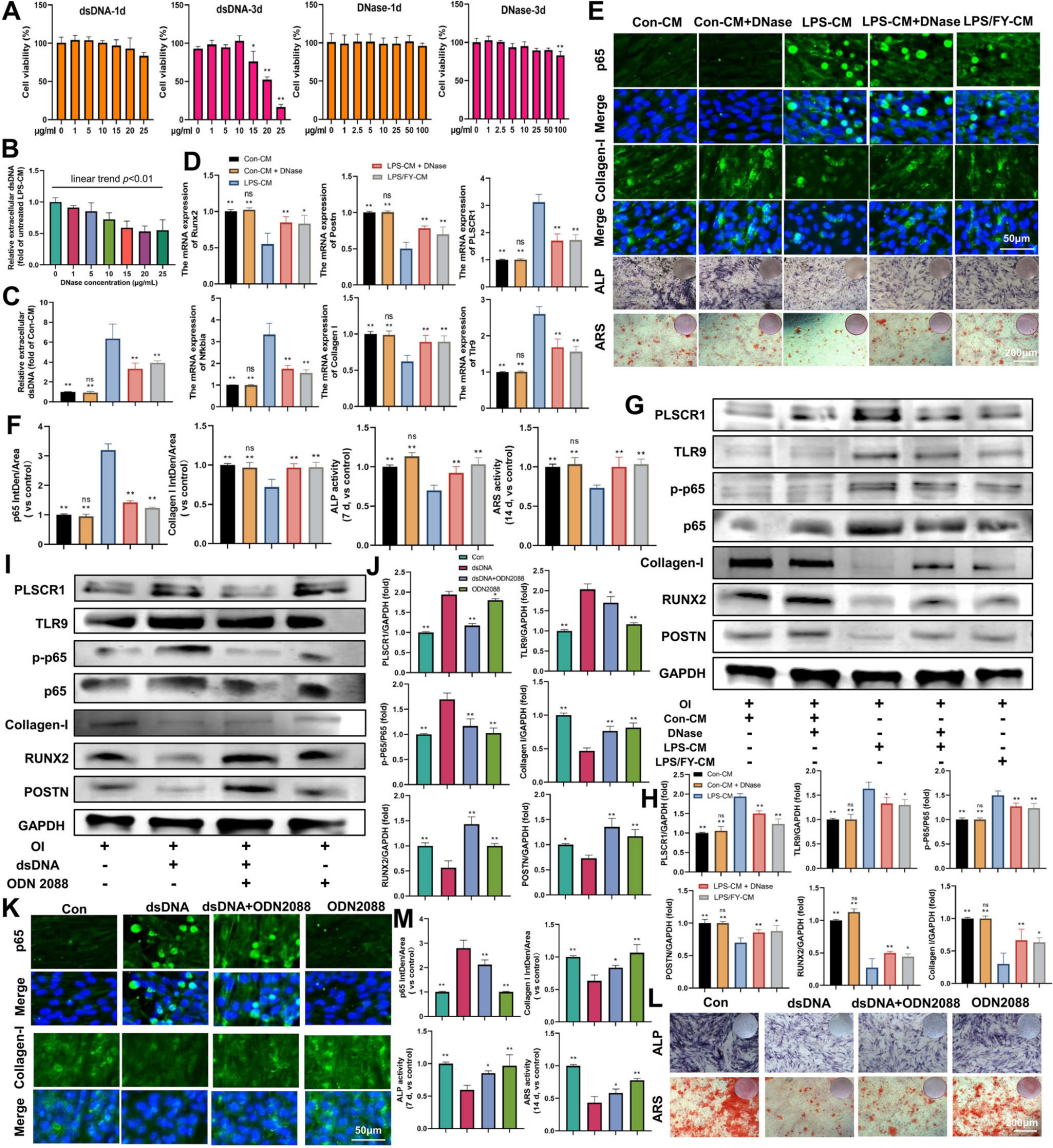
DNase treatment and separate dsDNA challenge support extracellular‐DNA involvement in osteogenic impairment. (A) rBMSC viability after DNase or dsDNA exposure on days 1 and 3. (B) DNase concentration‐dependent changes in extracellular dsDNA. (C) dsDNA in conditioned‐medium groups. (D) Runx2, Postn, Plscr1, Nfkbia, Col1a1 and Tlr9 transcripts. (E, F) p65/Collagen‐I immunofluorescence, ALP/ARS staining and quantification. (G, H) PLSCR1, TLR9, p65/p‐p65, Collagen‐I, RUNX2 and POSTN immunoblots and quantification. Groups: Con‐CM, Con‐CM+DNase, LPS‐CM, LPS‐CM+DNase and LPS/FY‐CM under osteogenic induction. DNase I: 10 µg/mL in CM, 37°C for 30 min before 1:1 dilution with induction medium. LPS/FY‐CM was collected after macrophage pretreatment with 5% FY serum. (I, J) Immunoblots and quantification after separate calf‐thymus dsDNA stimulation (final 5 µg/mL) with/without ODN2088; this was not an add‐back to DNase‐treated CM. (K–M) Corresponding immunofluorescence, ALP/ARS staining and quantification. Mean ± SD; n = 3 independent experiments. One‐way ANOVA with Dunnett comparisons: ^*^ *p* < 0.05, ^**^ *p* < 0.01 versus LPS‐CM (C–H) or OI+dsDNA (I–M). Con‐CM versus Con‐CM+DNase: unpaired t‐test; ns, not significant. Scale bars: see the corresponding images in the main text. Con‐CM, unstimulated macrophage‐conditioned medium; LPS‐CM, LPS‐stimulated macrophage‐conditioned medium; OI, osteogenic induction; ODN2088, TLR9 antagonist.

### 3.8 Pharmacological Intervention Supports TLR9/NF‐κB Involvement in FY‐Associated Osteogenic Protection

The TLR9 agonist ODN1826 and antagonist ODN2088 were used to assess pharmacological involvement of the pathway.

1. Effect of TLR9 activation alone on the osteogenesis of normal rBMSCs. CCK‐8 showed that ODN1826 and ODN2088 were not cytotoxic within the tested concentrations, and 5 µg/mL was selected (Figure 8A). Under a normal induction system, ODN1826‐mediated TLR9 activation alone significantly decreased ALP activity and inhibited ARS mineralization (Figure 8B, quantification in Figure 8C); immunofluorescence showed that it enhanced p65 nuclear translocation and downregulated Collagen‐I, indicating that TLR9 activation can activate NF‐κB and inhibit osteogenic matrix synthesis (Figure 8B).
2. TLR9 intervention modulates FY‐associated protection against inflammatory osteogenic injury. An inflammatory‐microenvironment osteogenic‐injury model was constructed with LPS‐CM. Immunofluorescence showed that LPS‐CM significantly increased p65 nuclear activation and decreased Collagen‐I, and 5% FY attenuated this phenotype; when combined with ODN1826, FY's inhibition of NF‐κB and its promotion of osteogenic matrix were both partially offset, whereas ODN2088 alone mimicked the protective effect of FY (Figure 8D, quantification in Figure 8E). That is, TLR9 activation attenuated, and TLR9 inhibition reproduced FY's anti‐inflammatory and osteogenesis‐promoting effects, indicating that the TLR9/NF‐κB pathway is functionally involved in this regulation.
3. Molecular readouts of TLR9/NF‐κB signaling and osteogenesis. The qPCR panel measured Tlr9, Nfkbia, Col1a1, Runx2 and Postn (Figure 8F). LPS‐CM was associated with higher inflammatory‐pathway readouts and lower osteogenic‐gene expression, whereas FY treatment shifted these readouts toward the OI condition. Western blots assessed PLSCR1, TLR9, p65/*p*‐p65, Collagen‐I, RUNX2 and POSTN (Figure 8G,H). The ODN1826 and ODN2088 treatment patterns, considered together with the functional assays, supported TLR9/NF‐κB involvement. The magnitude of the responses differed between readouts, and statistically supported pairwise differences are indicated in the panels. PLSCR1 protein expression changed alongside the pathway‐associated markers, supporting its description as a candidate upstream regulator rather than a functionally established mediator.
4. Functional osteogenic outcomes. LPS‐CM inhibited 7‐day ALP activity and 14‐day mineralization, and FY attenuated both effects. ODN1826 co‐treatment reduced this protection, while ODN2088 improved ALP activity and mineralization (Figure 8D,E). Together with the molecular readouts, these findings support functional involvement of TLR9/NF‐κB in FY‐associated protection against macrophage‐conditioned osteogenic impairment.

**FIGURE 8:**
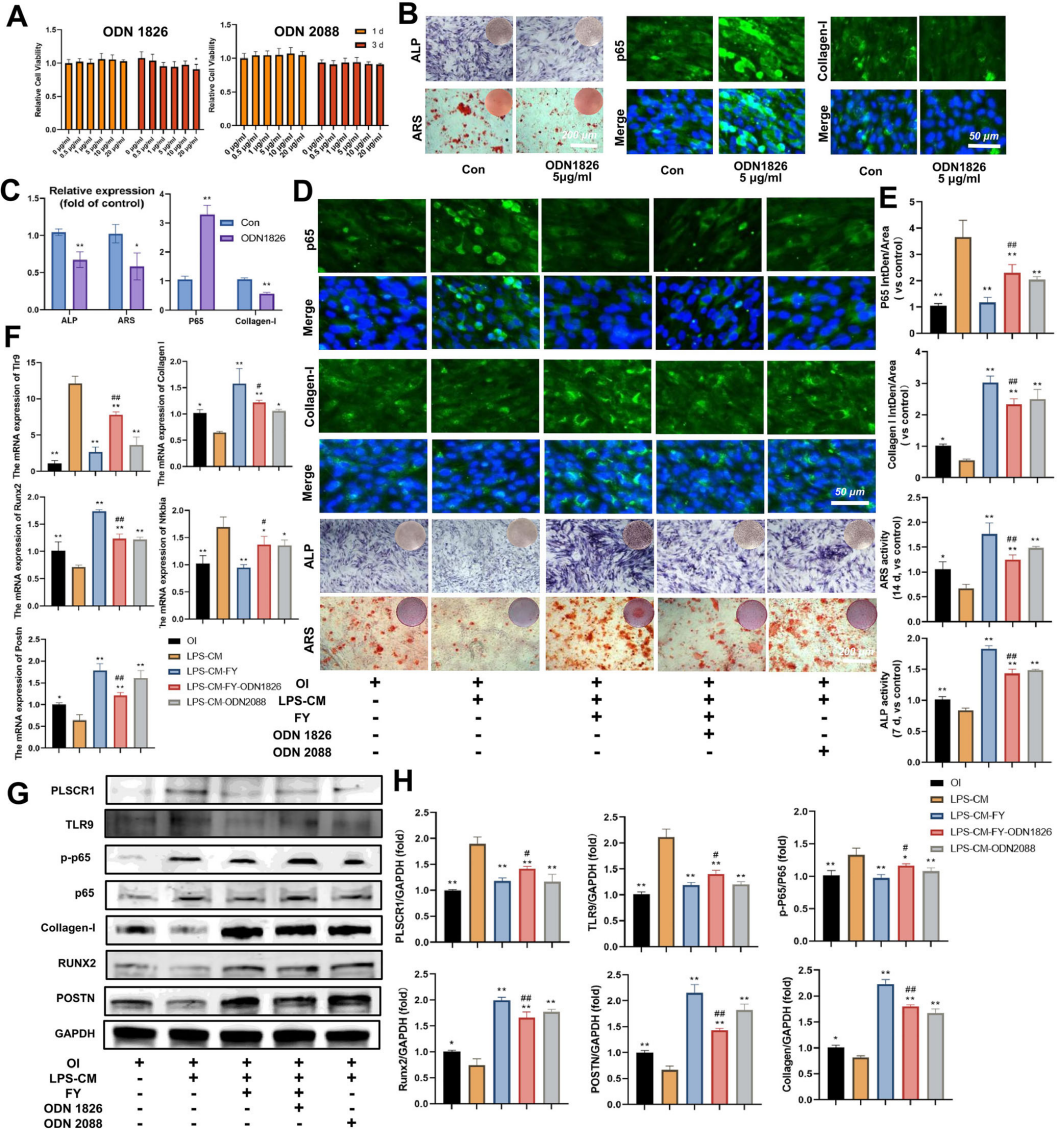
Pharmacological TLR9 modulation supports pathway involvement in FY‐mediated osteogenic protection. (A) ODN1826/ODN2088 viability screening. (B, C) ALP/ARS staining and p65/Collagen‐I immunofluorescence with quantification after ODN1826 (5 µg/mL) treatment. (D, E) The same readouts in OI, OI+LPS‐CM, OI+LPS‐CM+FY, OI+LPS‐CM+FY+ODN1826, and OI+LPS‐CM+ODN2088 groups. FY denotes 5% medicated serum added directly to rBMSCs. (F) TLR9, Col1a1, Runx2, Nfkbia, and Postn transcripts. (G, H) PLSCR1, TLR9, p65/p‐p65, Collagen‐I, RUNX2 and POSTN immunoblots and quantification. Mean ± SD; n = 3 independent experiments. Multi‐group comparisons: one‐way ANOVA with Tukey tests. ^*^ *p* < 0.05, ^**^ *p* < 0.01 versus LPS‐CM; #p < 0.05, ##p < 0.01 versus LPS‐CM+FY; in B–C, stars denote comparisons with the OI control. ns, not significant. Scale bars: see the corresponding images in the main text. OI, osteogenic induction; LPS‐CM, LPS‐stimulated macrophage‐conditioned medium; ODN1826/ODN2088, TLR9 agonist/antagonist.

### 3.9 In Vivo TLR9 Activation Offsets FY's Anti‐inflammatory and Pro‐osteogenic Effects at the Fusion Site

We performed a 2 × 2 pharmacological intervention in the rat XLIF model, with XLIF, H‐FY, ODN1826, and H‐FY+ODN1826 groups (n = 6 each). H‐FY was given orally at 4.5 g/kg/day in two divided doses, and ODN1826 was administered intraperitoneally at 100 µg per rat on postoperative days 1, 4, 7, 10, and 13; tissue was collected at 2 weeks (Figure 9A and Section 2.15.2). TLR9 immunostaining was lower with H‐FY and higher with ODN1826, with the combination group intermediate (Figure 9C, quantification in Figure 9F). ODN1826 co‐administration attenuated the FY‐associated reduction in F4/80^+^iNOS^+^ macrophage infiltration and *p*‐p65 and the increase in OPN, RUNX2, and Collagen‐I (Figure 9D–F). Across animals, *p*‐p65 and Collagen‐I were negatively correlated (Pearson r = −0.74; Spearman r = −0.85; *p* < 0.0001; Figure 9F). These findings support involvement of TLR9/NF‐κB in early inflammatory and osteogenic responses to FY in vivo. Because the intervention endpoint was 2 weeks and the agonist was administered systemically, this experiment does not establish dependence of final bony fusion on TLR9 or identify the responsible cell population.

**FIGURE 9:**
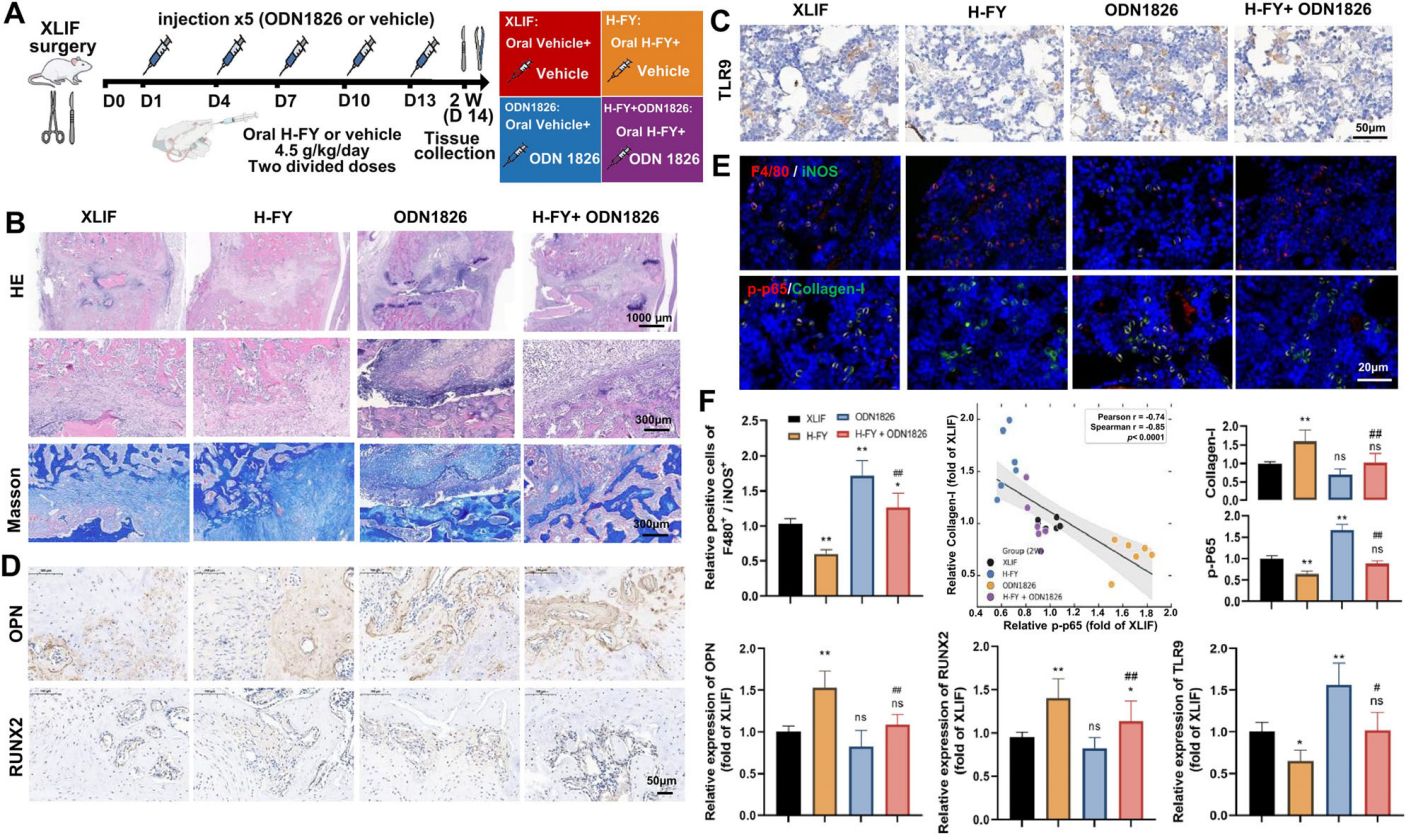
In vivo TLR9 activation modulates FY‐associated inflammatory and osteogenic responses at 2 weeks. (A) Treatment schedule: H‐FY, 4.5 g/kg/day orally in two equal divided administrations; ODN1826, 100 µg/rat intraperitoneally on days 1, 4, 7, 10 and 13; harvest on day 14. (B) H&E and Masson staining. (C) TLR9 immunohistochemistry. (D) OPN and RUNX2 immunohistochemistry. (E) F4/80/iNOS and p‐p65/Collagen‐I dual immunofluorescence. (F) Quantification of OPN, RUNX2, TLR9, F4/80+iNOS+ cells, p‐p65 and Collagen‐I, and Pearson/Spearman correlations between p‐p65 and Collagen‐I. Mean ± SD; n = 6 animals/group. Two‐way ANOVA (FY × ODN1826) with Tukey comparisons: ^*^ *p* < 0.05, ^**^ *p* < 0.01 versus XLIF; #p < 0.05, ##p < 0.01 versus H‐FY; ns, not significant. Scale bars: see the corresponding images in the main text. XLIF, operated vehicle control; H‐FY, high‐dose FY; ODN1826, TLR9 agonist. Hash symbols denote pairwise comparisons. The 2‐week endpoint assesses early tissue responses.

### 3.10 Human Nonunion Single‐Cell Data Support an Altered Inflammatory Myeloid‐Cell State

Secondary analysis of the human nonunion single‐cell dataset GSE217792 identified an altered myeloid‐cell expression pattern (Figure 10A). Relative to non‐fractured controls, PLSCR1 (*p* = 0.032) and IL6 (*p* = 0.037) were increased in the monocyte/macrophage population (Figure 10B). TLR9 transcripts were below the detection level, and NFKB1 and RELA did not differ significantly (Figure 10C). These findings support the relevance of an altered inflammatory myeloid‐cell state in human nonunion, but do not directly validate a dsDNA–TLR9/NF‐κB mechanism or demonstrate pathway activation in human BMSCs. The analysis involved a small case–control cohort without FY intervention and therefore does not establish clinical efficacy of FY.

**FIGURE 10:**
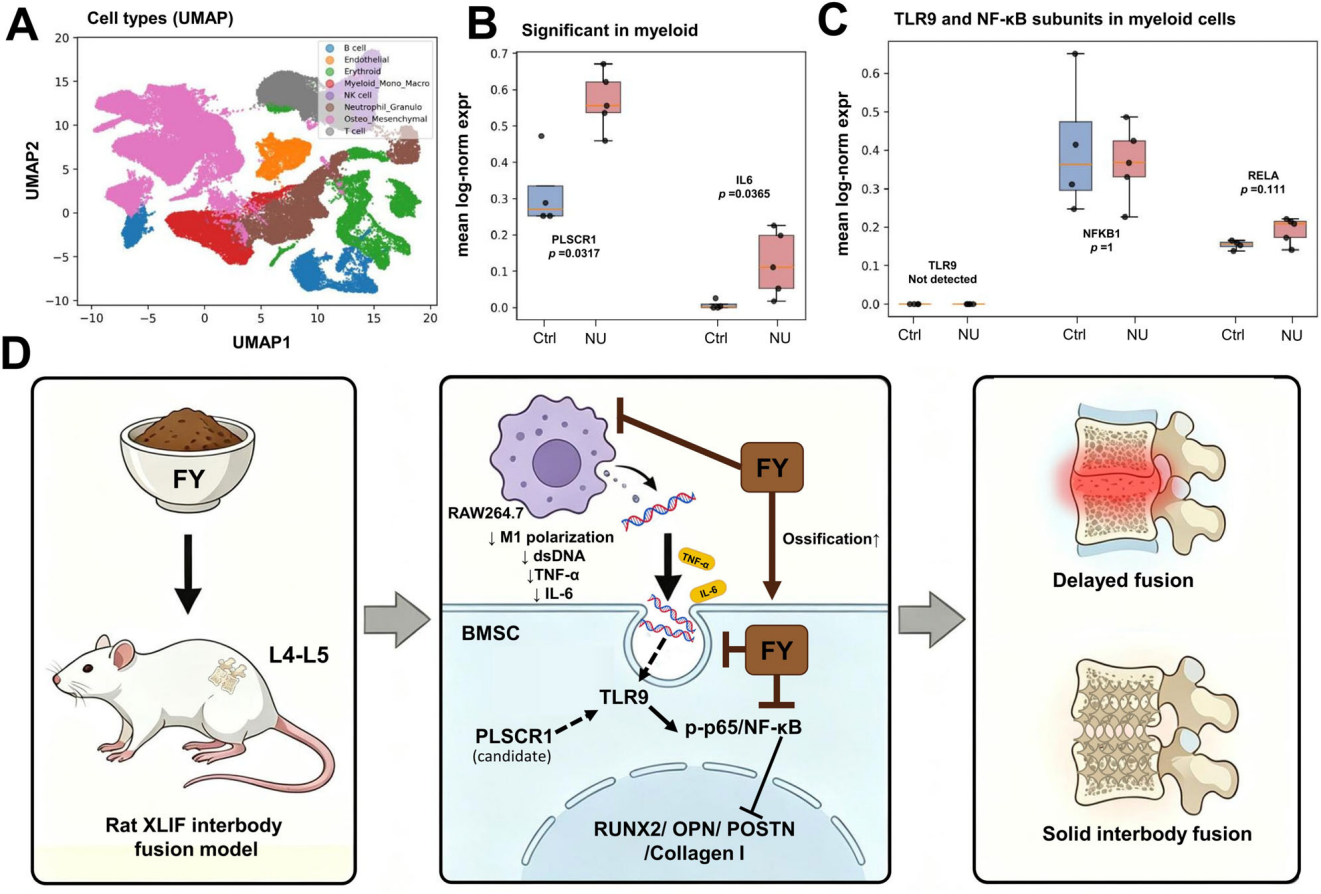
Human nonunion myeloid‐cell expression and a proposed model of FY action. (A) Single‐cell UMAP of control and nonunion samples. (B) Myeloid‐cell PLSCR1 and IL6 expression. (C) TLR9, NFKB1, and RELA expression. The sample was the statistical unit (4 controls, 5 nonunion cases); Mann–Whitney U tests, with *p*‐values shown. TLR9 was below detection in the analyzed population. (D) Proposed model linking reduced macrophage inflammatory output and extracellular‐DNA‐associated TLR9/NF‐κB signaling to improved BMSC osteogenesis. PLSCR1 is a candidate regulator; direct constituent binding and bidirectional cell interaction were not established. FY also promoted osteogenesis under non‐inflammatory conditions. Ctrl, control; NU, nonunion; UMAP, uniform manifold approximation and projection.

### 3.11 Body‐Weight and Organ‐Histology Findings Support Eight‐Week Tolerability

Body weight was followed in the 8‐week XLIF cohort (n = 6 per group), and the heart, liver, spleen, lungs, and kidneys were examined by H&E staining at the endpoint. All four groups showed an initial postoperative decrease in weight followed by recovery and broadly similar weight trajectories. No overt histopathological abnormalities were observed in the examined organs of FY‐treated animals relative to XLIF controls. Together with the cell‐viability findings (Figure 6B), these observations support tolerability under the tested conditions. They do not constitute a comprehensive assessment of systemic toxicity or establish safety beyond 8 weeks.

## 4 Discussion

### 4.1 Animal Efficacy and Complementary Osteogenic and Anti‐Inflammatory Effects

FY improved bone formation and microarchitecture in the rat XLIF fusion region, with dose‐related trends in selected outcomes and more developed bony bridging at the later endpoint (Figures 1 and 2). These findings establish a preclinical efficacy basis for investigating FY as an adjunct to interbody fusion. The interbody grafting and fixation model places repair within the intervertebral space, an anatomically relevant setting for this question [8, 36]. Although several H‐FY outcomes approached those of the PTH group, the study was not designed to establish therapeutic equivalence.

The functional experiments support two complementary effects. FY‐medicated serum directly promoted rBMSC osteogenic differentiation under non‐inflammatory induction conditions (Figure 6). It also attenuated the impairment produced by inflammatory macrophage‐conditioned medium when applied to rBMSCs (Figure 8). At the macrophage level, FY pretreatment reduced M1‐associated markers and extracellular dsDNA, and subsequently collected conditioned medium was less inhibitory to osteogenesis (Figures 5 and 7). Thus, the data support both an effect on osteogenic cells and modulation of the inflammatory environment in which they differentiate. Their relative contributions to the in vivo fusion benefit remain unresolved.

This interpretation fits the osteoimmunological view that sustained inflammation can constrain bone repair [14, 24, 25]. Recent scaffold and hydrogel approaches likewise combine immune regulation with support for bone formation [17, 18, 19, 47, 48]. FY extends this rationale to an orally administered formulation evaluated in interbody fusion; the present study does not compare its efficacy directly with these biomaterial strategies. Reduced inflammatory readouts at early endpoints and improved structural repair at 4 and 8 weeks are consistent with an inflammatory contribution to recovery. However, the 2‐week intervention cohort and the 4‐ and 8‐week main‐study cohorts were independent, and the cross‐time summary is descriptive. Together with the direct osteogenic effects in Figure 6, these observations support complementary actions without establishing a mandatory sequence in which inflammation must first resolve before osteogenesis can occur.

### 4.2 Extracellular DNA Contributes to Macrophage‐Conditioned Osteogenic Impairment

The Figure 7 experiments strengthen the link between inflammatory macrophage‐conditioned medium and BMSC dysfunction. DNase treatment reduced extracellular dsDNA in LPS‐CM and attenuated its effects on osteogenic‐marker expression, ALP activity, and mineralization. Con‐CM+DNase provided a control for DNase exposure under non‐inflammatory conditions. In a separate experiment, calf‐thymus dsDNA at 5 µg/mL impaired osteogenesis, and the TLR9 antagonist ODN2088 attenuated this response. These complementary findings support extracellular DNA as a contributor to inflammatory osteogenic impairment and provide a functional basis for examining DNA‐sensitive signaling in BMSCs. This interpretation is consistent with studies linking extracellular DNA to inflammatory responses, including TLR9‐associated signaling in other disease settings [30, 31, 32, 33].

The conditioned‐medium design examines directional effects from macrophages to BMSCs. Washing RAW264.7 cells after LPS exposure and collecting fresh conditioned medium reduced direct carryover, while LPS/FY‐CM allowed assessment of the effects of FY pretreatment on the subsequent activity of the medium. The DNase and exogenous‐DNA experiments address related but distinct questions: depletion tests the contribution of extracellular DNA in conditioned medium, whereas the separate challenge tests whether added DNA can impair osteogenesis under the tested conditions. Neither identifies the endogenous DNA species or traces its cellular origin. Accordingly, the results support a DNA‐associated component of the macrophage‐conditioned response, with cytokines and other mediators also potentially contributing.

### 4.3 Pharmacological Evidence for TLR9/NF‐κB Pathway Involvement

The in vitro and in vivo interventions provide complementary functional evidence for TLR9/NF‐κB involvement (Figures 8 and 9). In rBMSCs exposed to LPS‐CM, ODN1826 attenuated FY‐associated osteogenic protection, whereas ODN2088 improved osteogenic readouts alongside reduced NF‐κB‐associated responses. In the added 2‐week XLIF experiment, systemic ODN1826 co‐administration attenuated the FY‐associated reduction in inflammatory infiltration and p‐p65 and the increase in osteogenic markers. These findings support pathway involvement in FY‐associated protection across the cell and early tissue experiments. The in vivo endpoint addresses early inflammatory and osteogenic responses; it does not establish the pathway dependence of completed fusion.

TLR9 should be interpreted in its biological context. Global TLR9 deficiency has been associated with osteoclastic bone loss through gut‐microbiota‐associated systemic inflammation [49], while sustained NF‐κB activation can impair osteoblast differentiation and bone formation [50, 51]. The present results concern pharmacological modulation during inflammatory osteogenic injury, rather than the effects of removing TLR9 throughout the organism. PLSCR1 expression changed alongside the pathway‐associated readouts. Previous work demonstrated a role for PLSCR1 in TLR9‐mediated type I interferon production in plasmacytoid dendritic cells [28], providing a rationale for considering it a candidate upstream regulator here. That role has not been functionally established in the BMSC or fusion models.

The omics and docking analyses provided a basis for candidate selection. The BMSC RNA‐seq comparison contrasted FY‐treated osteogenic cultures with LPS‐CM‐exposed cultures and therefore nominated pathways without directly testing transcriptomic rescue of inflammatory injury. Docking identified predicted constituent–protein interactions for subsequent investigation. Reports on natural compounds such as tanshinone IIA and dehydrocostus lactone provide additional context for modulation of macrophage and Toll‐like receptor‐associated inflammatory signaling [52, 53, 54], and TLR9‐related effects have been investigated for other natural compounds in distinct disease models [55]. These observations do not identify the active constituent or direct target of FY. Other reported routes to improved osteogenesis or reduced inflammatory bone loss involve TLR2, HMGB1, or TRAF6‐associated signaling [56, 57, 58], emphasizing that the pharmacological evidence here supports TLR9/NF‐κB as a contributing pathway within a potentially broader response.

### 4.4 Human Relevance, Tolerability and Translational Limitations

The human nonunion analysis provides disease‐related context for the inflammatory findings. PLSCR1 and IL6 expression were higher in the analyzed myeloid‐cell population, whereas TLR9 was below detection and NFKB1 and RELA were not significantly different (Figure 10) [45]. This pattern supports an altered inflammatory myeloid‐cell state, without directly demonstrating the proposed mechanism in human BMSCs. Fracture nonunion and lumbar interbody fusion also differ in anatomy and clinical setting. At the tissue level, the convergence of inflammatory and ECM‐related pathways in our analyses is compatible with the established roles of focal adhesion and integrin signaling in osteogenic differentiation [59, 60]. Broader bone‐regeneration studies combining immune regulation, osteogenesis, or angiogenesis further support evaluating repair as a coordinated tissue response [61, 62, 63].

Body‐weight recovery and major‐organ histology in the 8‐week cohort showed no overt adverse findings associated with FY under the tested conditions. These observations support tolerability during the study period and provide a basis for further preclinical evaluation. For an orally administered herbal formulation, the relevant disposition questions concern absorption, metabolism, and elimination of its constituents. Constituent pharmacokinetics, active exposure levels, and compatibility with other perioperative treatments require investigation before clinical use can be inferred from these results.

Several limitations define the mechanistic interpretation. TLR9 was perturbed pharmacologically, without genetic manipulation or rescue, and PLSCR1 was assessed by expression rather than functional intervention. Systemic agonist administration does not identify the responding cell population, and the 2‐week intervention did not assess final fusion. The BMSC RNA‐seq design lacked an LPS‐CM+FY group, and docking was not followed by direct binding or cellular target‐engagement assays. DNase depletion was paired with a separate exogenous‐DNA challenge rather than a matched add‐back to DNase‐treated LPS‐CM; endogenous DNA identity, cellular origin, and contributions from other mediators remain unresolved. DNase was not removed before BMSC exposure, so its effects may also include continued extracellular DNA degradation during culture. The conditioned‐medium experiments did not assess reciprocal BMSC‐to‐macrophage regulation.

Further limitations concern model scope and translation. The temporal observations came from independent harvest cohorts and do not establish a longitudinal or causal sequence. Fusion was assessed by micro‐CT and histology without segmental biomechanical testing. The RAW264.7 and rBMSC models, medicated‐serum approach, and healthy‐rat experiments cannot fully represent human inflammatory conditions, constituent‐specific effects, or comorbidities that impair fusion. The human dataset was small and included no FY intervention. Finally, body weight and organ histology over 8 weeks do not establish comprehensive or longer‐term safety; laboratory measures of organ function, pharmacokinetic studies, and longer observation are needed. These boundaries guide the next stage of validation while preserving the demonstrated animal efficacy and the functional support for extracellular‐DNA‐associated TLR9/NF‐κB involvement.

## 5 Conclusion

FY improved lumbar interbody fusion and bone microarchitecture in rats. The functional data support complementary promotion of BMSC osteogenesis and attenuation of macrophage‐associated inflammatory injury. DNase treatment, a separate dsDNA challenge, and TLR9 pharmacological interventions support extracellular DNA and TLR9/NF‐κB signaling as contributors to these responses, with PLSCR1 remaining a candidate upstream regulator (Figure 10D). Together, these findings provide a preclinical basis for further evaluation of FY as an adjunct to interbody fusion.

## Author Contributions

H.G. conceived and designed the study, performed the experiments, analyzed the data, and wrote the original draft. H.W., S.L., W.W., and Y.G. contributed to the experimental work, data acquisition, and analysis. S.C., J.S., and J.A. assisted with data curation and validation. W.Z. supervised the project, acquired funding, and revised the manuscript. All authors reviewed and approved the final version of the manuscript.

## Funding

This study was supported by the 2026 In‐hospital Traditional Chinese Medicine and Integrative Chinese and Western Medicine Project of the Third Hospital of Hebei Medical University (No. ydsyzyy202601), the 2026 Hebei Provincial Higher Education Scientific Research Project (No. CXZX2026045), and the 2026 Government‐Funded Outstanding Clinical Medical Talent Project (No. ZF2026143).

## Conflicts of Interest

The authors declare that they have no known competing financial interests or personal relationships that could have appeared to influence the work reported in this paper.

## Supporting information




**Supporting File 1**: advs78444‐sup‐0001‐SuppMat.docx.

**Supporting File 2**: advs78444‐sup‐0002‐FigureTable.zip.

## Data Availability

The data that support the findings of this study are available in the supplementary material of this article.
