# Supplementary material for: Fuyuan Huoxue Decoction Promotes Lumbar Interbody Fusion in Rats: Role of Macrophage‐Driven Inflammation and the TLR9/NF‐κB Pathway
Source: Adv Sci (Weinh). 2026 Oct 29:e78444. Online ahead of print. doi: 10.1002/advs.78444 (PMC13720390; doi:10.1002/advs.78444)
Supplement: Supplementary file 1 [file ADVS-9999-e78444-s002.docx]

**Supporting Information**

**Fuyuan Huoxue Decoction Promotes Lumbar Interbody Fusion in Rats: Role of Macrophage-Driven Inflammation and the TLR9/NF-κB Pathway**

Hongwei Gao1, Han Wu1, Shaorong Li1, Weijian Wang1, Yijian Guo1, Shuai Cheng2, Jiayi Sun3, Jilong An4, Wei Zhang1*

1 Department of Spinal Surgery. The Third Hospital of Hebei Medical University, Shijiazhuang, People's Republic of China；

2 Laboratory of Sensory Neurobiology, School of Basic Medical Sciences, Hebei University, Baoding, People's Republic of China；

3 School of Basic Medicine, Hebei Medical University, Shijiazhuang, People's Republic of China；

4 Department of Spinal Surgery, Affiliated Hospital of Hebei University, Baoding, People's Republic of China；

*Corresponding author: Wei Zhang, E-mail: 37300332@hebmu.edu.cn

**This file includes:**

Supplementary Figure S1. Additional transcriptomic, network-pharmacology and single-cell analyses.

Supplementary Figure S2. Predicted docking poses of candidate constituents with TLR9 and p65.

Supplementary Figure S3. Eight-week body-weight and organ-histology assessment.

**Supplementary Figure S1. Additional transcriptomic, network-pharmacology and single-cell analyses.** (A–C) Rat fusion-site transcriptomics: PCA, differential-gene heatmap and selected osteogenic/inflammatory genes. (D–F) rBMSC transcriptomics: PCA, differential-gene counts and heatmap. (G) Network-pharmacology workflow. (H) Target-set overlap. (I) Pathway assignments of shared targets. (J) UMAP with human sample-group annotation. (K) Myeloid-cell proportions per sample (4 controls, 5 nonunion cases). PCA, principal component analysis; DEGs, differentially expressed genes.

**Supplementary Figure S2. Predicted docking poses of candidate constituents with TLR9 and p65.** (A,B) Reference-compound poses in TLR9 and p65 pockets. (C,D) Predicted two-dimensional interactions of C1, C2, C3, C8, C12 and reference compounds with TLR9 and p65, respectively. Chloroquine and helenalin were the respective reference compounds. Scores are AutoDock Vina predictions (kcal/mol), not measured affinities. PC, referencel compound.

**Supplementary Figure S3. Eight-week body-weight and organ-histology assessment.** (A) Representative heart, liver, spleen, lung and kidney H&E sections at 8 weeks. Scale bars are indicated in each panel. (B) Body-weight trajectories through postoperative day 56. Mean ± SD; n = 6 animals/group from the 8-week main-study cohort. ns, no significant difference for the indicated comparisons. XLIF, operated vehicle control; L-FY/H-FY, low-/high-dose FY; PTH, teriparatide. These endpoints assess tolerability during the observation period.
